# Baked sweetpotato textures and sweetness: An investigation into relationships between physicochemical and cooked attributes

**DOI:** 10.1016/j.fochx.2023.101072

**Published:** 2023-12-15

**Authors:** Matthew C. Allan, Suzanne D. Johanningsmeier, Mariam Nakitto, Osvalda Guambe, Modesta Abugu, Kenneth V. Pecota, G. Craig Yencho

**Affiliations:** a USDA-ARS, SEA, Food Science and Market Quality and Handling Research Unit, 322 Schaub Hall, North Carolina State University, Raleigh, NC 27695, USA; b International Potato Center (CIP-SSA), Plot 47 Ntinda II Road, PO Box 22247, Kampala, Uganda; c International Potato Center (CIP-MOZ), Av. FPLM 2698, PO Box 2100, Maputo, Mozambique; d Department of Horticultural Science, North Carolina State University, Raleigh, NC 27695, USA

**Keywords:** Sweet potato, Sweetness, Texture, Sensory, Ipomoea batatas, Texture profile analysis

## Abstract

Sweetpotato varieties vary greatly in perceived textures and sweetness. This study identified physicochemical factors that influence these attributes in cooked sweetpotatoes. Fifteen genotypes grown on three plots were baked and evaluated by a trained descriptive sensory analysis panel for sweetness and 13 texture attributes. Mechanical parameters were measured by texture profile analysis (TPA); and composition (starch, cell wall material, sugar contents), starch properties (thermal, granule type ratios, granule sizes), and amylase activities were characterized. TPA predicted fracturability and firmness well, whereas starch and sugar contents, B-type starch granule ratio, and amylase activities influenced prediction of mouthfeel textures. Sweetness perception was influenced by perceived particle size and sugar contents; and maltose generation during baking was highly correlated with raw sweetpotato starch content. These relationships between physicochemical sweetpotato properties and baked textures and sweetness could benefit breeders and processors in selecting biochemical traits that result in consumer preferred products.

## Highlights

• Instrumental hardness was highly correlated with sensory firmness.
• Sensory textures were affected by multiple physicochemical properties of sweetpotato.
• Starch granule size was not a strong predictor of baked sweetpotato textures.
• Perceived particle size modulated the impact of sugar contents on sweetness.
• Maltose production during baking was highly correlated with starch content.

## 1 Introduction

Sweetpotato (*Ipomoea batatas* L.) is a starchy root crop that is consumed around the world, with approximately 90 million tonnes being produced globally. Sweetpotato is a good source of essential vitamins, minerals, and fiber and is an important food security crop with 25 to 30 % of the global production being grown in “low income food deficit countries” (FAOSTAT., 2023, Loebenstein, 2009).

New sweetpotato varieties are being continuously developed by sweetpotato breeding programs around the world for improved agronomic traits, disease resistance, value-added product functionality, and improved nutrition. New varieties must also meet consumer-preferred culinary attributes to become widely adopted. However, meeting consumer preferences is a challenge because sweetpotatoes are a diverse crop with various flesh colors (white, cream, yellow, orange, or purple), unique aromas (e.g., chestnut, potato-like, caramel, pumpkin), wide-ranging sweetness levels, and unique textural properties (e.g., moist, dry, mealy, watery, cohesive). Preferences for these various traits differ between countries and even within regions of nations. In the United States, particularly in the Southern US, moist, sweet, orange-fleshed sweetpotatoes are traditionally preferred (Leksrisompong, Whitson, Truong, & Drake, 2012). In contrast, the orange-fleshed varieties were disliked due the “watery” texture in a South African study (Laurie et al., 2013), and with consumers preferring firm and dry textures. Similarly, sweetpotato genotypes that were sweet with firm and dry textures were preferred in Uganda (Mwanga et al., 2020). Kays, Wang, and McLaurin (2005) also reported varying sweetpotato preferences based on varietal sugar contents and country of origin. For example, American consumers preferred “very high” sweetness, Nigerian consumers preferred “high” sweetness, and Japanese and Chinese consumers preferred “moderate” sweetness in their sweetpotatoes. Understanding relationships between the physicochemical properties of raw sweetpotatoes and the sensory attributes of cooked sweetpotatoes will assist breeders and processors in making selections to meet these varying consumer preferences.

Cooked sweetpotato and sweetpotato product textures and sweetness have been previously associated with composition, amylase activities, and starch properties. In general, higher starch/dry matter contents, lower amylase activities, and higher gelatinization temperatures tend to lead to dry and firm sweetpotatoes; whereas higher sugar contents and β-amylase activities have been associated with sweet, soft, and moist sweetpotatoes (Allan et al., 2023, Kitahara et al., 2017, Sato et al., 2018, Walter et al., 1975, Yoon et al., 2018). Cooking methods can also impact sweetpotato texture and sweetness (Barkley et al., 2017, Nwosisi et al., 2019, Zhang et al., 2023). During cooking, starch gelatinizes, thermally activated amylases catalyze the hydrolysis of starch into dextrins (α-amylase) and maltose (β-amylase), and cell wall polymers begin to soften (Binner et al., 2000, Takahata et al., 1994). Sweetpotato textures can be partially predicted by mechanical texture analyses, such as the Texture Profile Analysis (TPA), but many sweetpotato textures were less than 50 % explained using this method (Truong, Walter, & Hamann, 1997). Similarly, Nakitto et al. (2022) reported that mechanical analysis could only predict about 65 % of the total variation in sweetpotato firmness. This study will utilize the most recently developed sweetpotato lexicon (Nakitto et al., 2022) and advances in starch chemistry analyses to investigate the relationships between physicochemical sweetpotato properties and the complex textures of baked sweetpotato.

We hypothesize that (A) starch thermal properties and amylase activities affect baked sweetpotato textures by influencing the changes in starch components during baking. Thus, predictive models for texture attributes will be improved by the inclusion of these physicochemical sweetpotato properties. Furthermore, we posit that (B) variations in the textures affect perceived sweetness of baked sweetpotatoes due to differences in the rate of release of sweet stimuli and that models for sweetness will improve when textural properties are included along with sugar contents. Therefore, our objective was to evaluate the textures and sweetness of a diverse set of sweetpotatoes; measure sweetpotato mechanical parameters; characterize the compositions, amylase activities, and starch properties; and investigate factors associated with baked sweetpotato textures and sweetness using both univariate and multivariate models. Identification of key predictors of sweetness and textures will help breeders and processors select genotypes that meet varying consumer preferences as well as better understand the effects of physicochemical sweetpotato properties on baked sweetpotato sensory attributes.

## 2 Materials and methods

### 2.1 Raw materials

Fifteen sweetpotato clones and/or released varieties (Covington, Beauregard, NC13-1027, Bonita, NCDM04-0001, Kokei No. 14 (Japanese), Dimbuka Bukulula, O'Henry, NCP16-0046, NCP16-0095, NC15-0633, NC16-0613, NC17-0331, NCMC16-0298 and NCP13-0315) were grown in 3 replications: 2 plots at the North Carolina Department of Agriculture and Consumer Services (NCDA&CS) Horticultural Crops Research Station in Clinton, NC and 1 plot at the North Carolina State University (NCSU) Cunningham Research Station in Kinston, NC. These plots were planted on June 10th, June 18th, and July 1st, 2021, then harvested in October 2021 – 120 to 140 days after planting. Harvested storage roots (hereafter referred to as roots) were cured for 7 days at 27˚C and 85 % RH then stored at 14˚C and 60 % RH until June 2022.

### 2.2 Sweetpotato baking

Six to nine roots with uniform size and shape were selected from each genotype and plot. Roots were washed, individually wrapped in aluminum foil, placed on a baking sheet with > 5 cm of separation between roots, and baked for 60–75 min at 188 ˚C. After baking, roots were cooled for ∼ 5 min at ambient temperature and cut lengthwise (longitudinal with the vascular system). One half of each root was set aside for laboratory analyses and the other half prepared for sensory analysis. After mechanical texture analysis, the halves saved for laboratory analysis were combined into a composite sample, taking care to use approximately the same amount from each root, pureed using a Robot Coupe food processor (Ridgeland, MS, USA), then frozen at −20˚C until further analysis.

### 2.3 Sensory analysis

#### 2.3.1 Sample preparation

Approximately 2 cm cubes were cut from the baked sweetpotato halves allocated for sensory analysis. One cube from each root of the sample set was placed into individual 4 oz souffle cups labeled with a random three-digit code such that each panelist would receive a sample that was representative of all the roots prepared for each genotype and plot. Cups were capped and incubated at 40˚C for 0.5 to 1 h before sensory evaluation.

#### 2.3.2 Sensory panel

A panel of 9 members, most of whom had more than 100 h of prior training in descriptive sensory analysis, were trained in six 3-hour training sessions for descriptive analysis of baked sweetpotatoes using a sweetpotato lexicon adapted from Nakitto et al. (2022). The panel rated sweetpotato samples for sweetness and 13 textural attributes: (first bite) fracturability, (compression) firmness, crunchiness, (chewdown) moisture in mass, mealiness, adhesiveness, moisture release, cohesiveness of mass, surface smoothness, particle size, rate of breakdown, fibrousness, and uniformity of texture. Descriptions and references for sweetness and textures are reported in the Appendix (Table A.1). For each day of analysis, panelists were provided with a marked Covington reference sample for calibration, which had agreed upon scores for the sensory attributes (Figure A.1). This was followed by monadic evaluation of 5 unique samples coded with a random 3-digit number. Ratings for each of the sensory attributes were recorded in Compusense 20 using intensity scales that ranged from 0 to 10 and were anchored with descriptive terms (Compusense Inc., Guelph, Ontario, Canada).

### 2.4 Texture profile analysis

Texture profile analysis (TPA) was conducting using the other half of the baked sweetpotato and followed protocols reported by Truong et al. (1997); Truong, Walter, and Bett (1998) with minor modifications. Briefly, longitudinal cylinders were cut from baked sweetpotato root halves using a #9 cork borer (2.25 cm diameter) then trimmed to 2.2 cm lengths. The TPA of each cylinder was collected using a Texture Analyzer TA.XT2 (Stable Micro Systems, Godalming, UK) with a 50 Kg load cell, TA-25 probe (5.1 cm diameter), and flat platform. TPA test conditions were 1.6 cm/s speed, compress 1.65 cm (75 % of original height) once triggered by contact with the sample, return to starting position, 5 s delay, then another 1.65 cm compression. Hardness, fracturability, adhesiveness, springiness, cohesiveness, gumminess, and chewiness values were calculated by the software using equations reported by Friedman, Whitney, and Szczesniak (1963). Where possible, two cylinders from each root were measured and texture properties were averaged as texture attributes of individual roots. Root textures were then averaged within a sample set composed of 6 to 9 roots as a representative texture for a given genotype and plot.

### 2.5 Compositional analyses

#### 2.5.1 Dry matter

Dry matter contents of raw and baked sweetpotatoes were measured using a modified AOAC 925.45 (AOAC, 1990) method. Frozen raw slices were broken into 2 to 5 mm pieces before analysis by crushing with a rubber mallet in polyethylene, resealable, 1-gallon sized bags. Since baked sweetpotato samples were already homogenous, a portion of the frozen slab was simply broken off. For both baked and raw sweetpotato samples, 5 g of frozen sweetpotato pieces were dried in 57 mm aluminum pans for 2 days at 60 ˚C and < 1 kPa with a slow purge of ambient air in an AT09 vacuum oven (Across International, Berkeley Heights, NJ, USA). The average of duplicate measurements was used as the moisture contents for each biological replicate.

### 2.6 Freeze drying

Five roots from each genotype and location replicate were washed, peeled, sliced 1.5 mm thick latitudinally using a Hobart FP150 continuous feed food processor (Hobart Corp., Troy, OH, USA), then frozen at −20 ˚C. Frozen raw slices and frozen baked sweetpotato purees were freeze dried with a Harvest Right Scientific Freeze Dryer (North Salt Lake, UT, USA) at < 65 Pa using the following protocol: −30 ˚C for 2 h at ambient pressure, −30 ˚C for 24 h, −15 ˚C for 12 h, −5 ˚C for 12 h, 10 ˚C for 12 h, and 25 ˚C for 12 h.

#### 2.6.1 Baked and raw sweetpotato sugar contents

Sugar contents of baked and raw sweetpotatoes were measured using the method reported by Allan and Johanningsmeier (2022) with slight modifications. One gram of freeze dried sweetpotato powder was weighed into 50 mL polyethylene centrifuge tubes, 7 mL of 70 ˚C 80 % ethanol was added, vortexed for 1 min, and centrifuged for 10 min at 6500 *g*. The supernatant was decanted into a 50 mL volumetric flask, extraction steps were repeated for a total of 3 extractions, then the volumetric flask was brought to volume. Two hundred microliters of extracts were dried for 30 min at 60˚C followed by no heating for another 30 min while at < 1 kPa in a Speed-Vac SPD1030 Integrated Vacuum Concentrator (Savant, ThermoFisher Scientific). Dried solids were reconstituted with 2 mL of water and passed through a 13 mm, 0.45 µm nylon syringe filter into HPLC vials. Sugars were separated isocratically with 200 mM NaOH at 1 mL/min on a Dionex™ CarboPac™ PA1 (4 × 250 mm) and guard column (4 × 50 mm) (ThermoFisher Scientific) at 30 ˚C then detected using an Antec Scientific Decade Elite (Zoeterwoude, Netherlands) PAD with a SenCell 2 mm Au HyRef reference cell. External standard curves of glucose, fructose, sucrose, and maltose were used to quantify sugar contents.

#### 2.6.2 Starch contents

Raw sweetpotato starch contents were measured using the remaining desugared alcohol insoluble solids from the sugar extraction and the Megazyme (Wicklow, IE) Total Starch Assay Kit (AA/AMG). Starch contents of raw sweetpotatoes were calculated accounting for raw moisture and sugar contents.

#### 2.6.3 Cell wall material contents

Cell wall material contents of raw sweetpotatoes were calculated by dry matter minus the starch and total sugar contents (Allan, Marinos, Johanningsmeier, Sato, & Truong, 2021).

#### 2.6.4 α and β amylase activities

The α-amylase and β-amylase activities of the freeze-dried raw sweetpotato powder were measured using the Megazyme α-Amylase Assay (Ceralpha Method) and β-Amylase Assay Kits (Betamyl-3) following the instructions from the manufacturer.

### 2.7 Starch characterization

#### 2.7.1 Starch extraction

Starch was extracted using a modified traditional potato starch extraction method (De Willigen, 1964). About 50 to 100 g of frozen raw sweetpotato slices and 200 mL of water were mixed for 1 min in a Waring Commercial Spice Grinder (Stamford, CT, USA). The mixture was passed through 2 layers of grade 60 cheesecloth to remove pulp and large particles. The filtrate was centrifuged at 200 *g* for 2 min in 250 mL centrifuge bottles. This low centrifugation force was used to collect starch at the bottom but keep much of the non-starch particulates (e.g., cell wall fragments) suspended. The supernatant was discarded then 200 mL of water was added to the starch, mixed vigorously for 10 to 20 s, centrifuged using the same conditions, and repeated for a total of 3 extraction cycles. Thirty mL of 95 % ethanol was added to the starch, mixed until the starch was completely suspended, then poured into 50 mL centrifuge tubes. Tube weights were equalized by adding additional 95 % ethanol where needed, mixed, and centrifuged for 5 min at 3,000 *g*. The supernatant was discarded then extracted starches were air dried for 3 days in a fume hood then finish dried at 40 ˚C for 2 h.

### 2.8 Thermal analysis

Starch thermal and data analyses are those previously reported in Allan et al. (2023). Briefly, 1:2 w/w starch to water slurries were equilibrated overnight, 10 to 15 mg of resuspended slurry was pipetted into 50 μL aluminum pans, hermitically sealed, then heated from 10 to 105 ˚C at 10 ˚C/min in a Perkin Elmer DSC 6000 (Waltham, MA, USA) that was calibrated with indium and tin. Thermograms were deconvoluted using “Interactive Peak Fitter” (O’Haver, 2021) run in MatLab R2022b (The MathWorks, Inc., Santa Clara, CA, USA). Starch thermal properties will be compared by peak temperatures and ratios of the B-, C-, and A-type starch granules, which are in order from lowest to highest gelatinization temperatures (Guo, Zhang, Bian, Cao, & Wei, 2020).

### 2.9 Starch granule size

Starch granule size distributions were measured using the same methods as reported in Allan et al. (2023). Briefly, particle sizes of starch slurries were measured using a Malvern Mastersizer 3000 with a Hydro EV wet dispersion attachment (Spectris, Egham, UK). Percent volume densities of starch granules were calculated using particles from 1 to 50 μm and the 1.3 g/cm^3^ density of hydrated starch (Dengate, Baruch, & Meredith, 1978).

### 2.10 Data analysis

All data analyses were performed in JMP Pro (v16.2.0, SAS, Cary, NC). Data distribution of inputs were initially screened for normality and skewness, and the sensory attributes of crunchiness and moisture release were omitted due to poor distributions. Texture profile analysis resilience values were ≈ 0 and thus left out, and adhesiveness was omitted since TPA is not the recommended method for this texture (Texture Technologies, 2015).

Principal component analysis (PCA) of sensory texture attributes was conducted and sweetpotato genotypes were plotted as a supplementary variable, which did not affect the PCA distributions of the textures. Sweetpotato samples were clustered based on sensory textures using the “K Means Cluster” application.

Univariate linear relationships between physicochemical or TPA attributes to sensory textures with R^2^ > 0.50 (p < 0.001) were noted and considered significant. The prediction strengths of these linear correlations were evaluated by the R^2^ and root average square error (RASE) of validation sets from a K-fold (K = 5) cross validation. Random forest multivariate models were conducted using the “bootstrap forest” platform with K-fold (K = 5) cross validation, and prediction accuracy was also compared using the R^2^ and RASE of validation sets. Factors in random forest models were considered significant if the factor contribution portion was > 10 %.

Structural equation modeling (SEM) was used to investigate factors affecting perceived sweetness. The SEM hypothesis structure was that individual sugars plus texture affect sweetness perception, and starch content and β-amylase activity affect both maltose content and texture. Textures evaluated in SEMs were particle size, mealiness, moisture in mass, cohesiveness, and combinations thereof. The SEM with particle size as the sole texture was the most appropriate model based on software provided fitting criteria (e.g., Akaike’s Information Criterion [AICc] and Bayesian Information Criterion [BIC]). Data were first standardized before SEM.

## 3 Results and discussion

### 3.1 Sensory analysis of baked sweetpotato textures

**Fig. 1:**
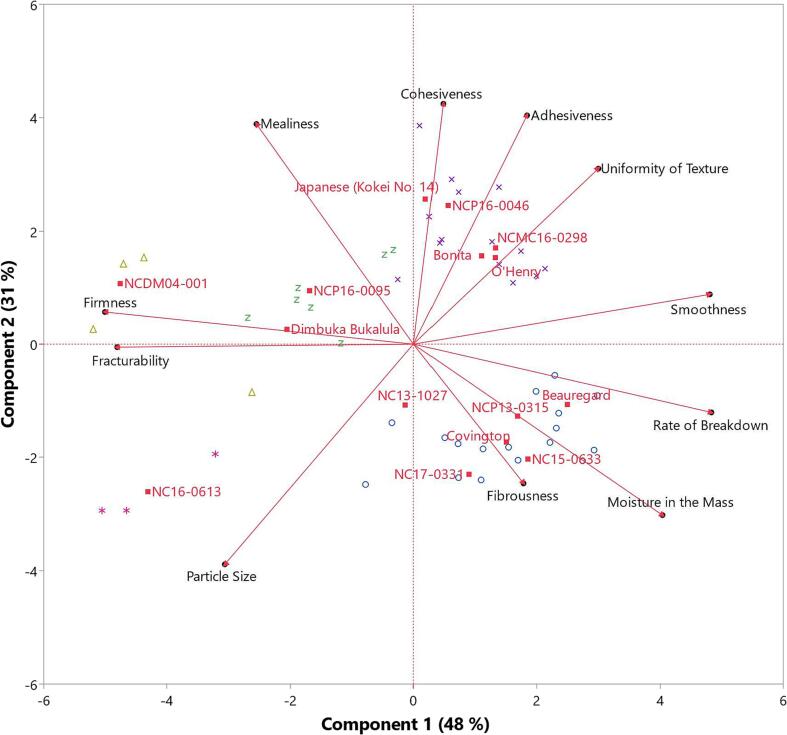
Principal component analysis of baked sweetpotato texture attributes (black dots). Clustering of observations are indicated by shared marker shape and color, and average values for sweetpotato genotypes are plotted as a supplementary variable (red boxes). (For interpretation of the references to color in this figure legend, the reader is referred to the web version of this article.)

The textural attributes of these sweetpotato genotypes were diverse and were grouped into 5 clusters based on their overall sensory texture profiles (Fig. 1). The first two principal components accounted for 79 % of the variability. Textures loading high on principal component 1 (48 %) were fracturability, firmness, smoothness, and rate of breakdown, whereas key textures for principal component 2 (31 %) were mealiness, adhesiveness, cohesiveness, and particle size (Table A.3). In Fig. 1, cluster 1 (blue ○) is comprised of sweetpotatoes that tended to be high in perceived moisture, fibrous, and low in mealiness and firmness. Cluster 1 was representative of the typical orange-fleshed sweetpotato varieties in the US, such as Beauregard and Covington. Cluster 2 (purple X) sweetpotatoes were high in cohesiveness, adhesiveness, uniformity of texture, and low in perceived particle size. Varieties in this cluster were the typical white/cream-fleshed varieties in the US such as Bonita, O’Henry, and Japanese. These first 2 clusters were similar to those observed in a previous study on baked sweetpotatoes (Leksrisompong et al., 2012). Cluster 3 (green Z) sweetpotatoes were higher in mealiness and firmness and lower in moisture in mass and represented by Dimbuka Bukalula, an African landrace sweetpotato variety (Mwanga et al., 2009). Sweetpotatoes in cluster 4 (yellow Δ) were similar to cluster 3 but even more firm and dry, including NCDM04-001, a β-amylase null genotype. The fifth cluster (pink *) was comprised of sweetpotatoes that were high in firmness and particle size and low in uniformity of texture, specifically the genotype NC16-0613, which had a unique uncooked-like texture similar to that observed in sweetpotato French fries prepared from genotype NC13-487 (Sato et al., 2018).

### 3.2 Predicting sweetpotato textures with texture profile analysis

The relationships between sweetpotato sensory texture attributes and TPA parameters were investigated. TPA is a high-throughput, routine analysis that has been previously correlated to cooked sweetpotato (Truong et al., 1997). TPA is also being currently used for screening genotypes in the CIP sweetpotato breeding programs in Uganda and Mozambique. Identification of TPA parameters that predict baked sweetpotato sensory textures will aid breeding programs and future sweetpotato texture studies as well as highlight any limitations of TPA to relate to human perception of sweetpotato texture.

The TPA profiles of the sweetpotatoes in this study varied greatly. For example, hardness values ranged from 354.8 to 4231.1 g and springiness values from 18 to 35 % (Table A.5). TPA fracturability was not measurable in very soft sweetpotatoes, but when quantified, it was highly correlated (R^2^ = 0.90) with hardness (Data not shown). Due to this co-linearity and missing values, TPA fracturability was not included in subsequent data analyses. TPA hardness values plus gumminess and chewiness (both are calculated using hardness) were also log_10_ transformed due to data skewness (Figure A.2). Log hardness was the best predictor for sensory fracturability (R^2^ = 0.74) and firmness (R^2^ = 0.89), and somewhat correlated with smoothness (R^2^ = 0.67). Log chewiness was the best predictor for sensory rate of breakdown (R^2^ = 0.83) and partially correlated with moisture in mass (R^2^ = 0.63) (Tables 1 and A.7). Other statistically significant correlations with sensory textures were observed but not notable. Sensory mealiness, adhesiveness, cohesiveness, particle size, fibrousness, and uniformity of texture scores were not well predicted (R^2^ < 50 %) by linear relationships with TPA parameters.

Random forest multivariate prediction models were used to investigate complex relationships between TPA and sensory textures, and the cross-validation prediction R^2^ and RASE values between the univariate and multivariate models were compared. Fracturability, firmness, smoothness, and rate of breakdown predictions using the best univariate model were similar to the multivariate models (Table 1), which suggests multivariate models do not account for more of the variance beyond these univariate relationships. The univariate relationship between log chewiness and moisture in mass was not robust, as the correlation of the cross validation was weak. About 50 % of the variance of moisture in mass and particle size was explained by the random forest models, but overall, these textures were still largely unexplained by TPA.

**Table 1:** Univariate and multivariate sensory texture prediction models using texture profile analysis and physicochemical properties (composition, amylase activities, and starch properties). The univariate predictor with the highest R^2^ and the direction of the relationship (positive (+) or negative (-)) are reported along with the univariate and multivariate prediction R^2^ (Val. R^2^) and root average square error (RASE) from a K-fold cross validation (K = 5). Univariate relationships and multivariate predictions with R^2^ > 0.50 are reported.

	**Texture Profile Analysis**						**Physicochemical Properties**					
	Univariate				Multivariate		Univariate				Multivariate	
**Sensory Texture**	Predictor	R^2^	Val. R^2^	RASE	Val. R^2^	RASE	Predictor	R^2^	Val. R^2^	RASE	Val. R^2^	RASE
Fracturability	Log Hard.	0.74 (+)	0.68	0.85	0.67	0.79	NA				NA	
Firmness	Log Hard.	0.89 (+)	0.86	0.65	0.78	0.73	NA				0.57	1.08
Moisture in the Mass	Log Chew.	0.63 (-)	0.25	1.16	0.51	1.01	Starch	0.64 (-)	0.43	0.89	0.66	0.92
Mealiness	NA				NA		Starch	0.68 (+)	0.56	0.74	0.74	0.55
Adhesiveness	NA				NA		NA				0.54	0.45
Cohesiveness	NA				NA		NA				0.61	0.76
Smoothness	Log Hard.	0.67 (-)	0.58	0.98	0.57	0.86	NA				NA	
Particle Size	NA				0.50	0.97	B-Type Peak	0.58 (-)	0.59	0.74	0.52	0.96
Rate of Breakdown	Log Chew.	0.83 (-)	0.80	0.82	0.75	0.87	NA				0.54	1.24
Fibrousness	NA				NA		NA				NA	
Uniformity of Texture	NA				NA		NA				NA	

NA = not applicable; R^2^ < 0.50.

TPA could predict sensory fracturability, firmness, and rate of breakdown, which these relationships are logical because they are experienced in a similar manner as the TPA method. The relationships between mouthfeel textures and TPA were not clearly related, and any statistical significance was possibly a result of being confounded with the well-predicted textures (Table A.4). In agreement with Nakitto et al. (2022), TPA was limited to first bite and compression textures and could not predict sweetpotato mouthfeel textures such as mealiness, adhesiveness, cohesiveness, particles size, fibrousness, and uniformity of texture.

### 3.3 Sweetpotato compositions, starch properties, and amylase activities

Sweetpotato samples ranged in 15.5 to 40.3 % dry matter, 3.5 to 26.3 % starch, 4.9 to 12.2 % cell wall material, 1.3 to 23.5 mg/g glucose, 0.4 to 14.0 mg/g fructose, 11.6 to 63.0 mg/g sucrose, 0.65 to 118.6 Ceralpha® U/100 g α-amylase, and 6.5 to 933.3 Betamyl-3® U/100 g β-amylase (Tables A.8 and A.9, Data Repository). Maltose was below the detections limits in all of the raw sweetpotatoes, but baked sweetpotatoes contained maltose concentrations ranging from 0 to 119.2 mg/g. Isolated sweetpotato starch properties were also diverse, ranging from 0 to 66.7 % B-type starch with peak temperatures from 56.8 to 72.3˚C, 16.1 to 76.6 % C-type starch with peak temperatures from 61.4 to 78.7˚C, and 10.6 to 76.9 % A-type starch with peak temperatures from 68.5 to 84.5˚C. Starch granules sizes in the 10th, 50th, and 90th percentiles ranged from 2.9 to 9.9, 8.7 to 17.7, and 16.6 to 32.4 μm, respectively (Tables A.8 and A.9, Data Repository). The sites where sweetpotato replicates were grown did not significantly influence sweetpotato compositions and starch properties (data not shown).

This was a diverse set of sweetpotato genotypes with a wide range of physicochemical properties that encompass most sweetpotato varieties. For example, the dry matter and starch contents ranged from an exceptionally low dry matter in NC17-0331 (16.6 % DM and 4.3 % starch) to high dry matter in NCDM04-001 (37.2 % DM and 22.3 % starch). CIP reported the dry matter and starch contents of 106 sweetpotato clones, and the means and ranges were from 19.9 to 45.4 % dry matter and from 11.1 to 33.5 % starch (Brabet et al., 1999). Our genotypes tended to be lower in dry matter but the high dry matter sweetpotato type was represented by 4 genotypes with dry matters > 30 % (Table A.8). The amylase activities were also diverse and ranged beyond the 1st and 3rd quartiles of α and β-amylase activities of stored sweetpotatoes (256 progeny for 2 years) reported by Amankwaah et al. (2023). Therefore, this set of sweetpotato genotypes was diverse and represented the range of most sweetpotato varieties.

#### 3.3.1 Predicting sweetpotato texture attributes from physicochemical sweetpotato properties

Raw sweetpotato composition, starch properties, and amylase activities were explored to identify physicochemical sweetpotato properties that influence baked sweetpotato textures. Dry matter and starch content were highly correlated (R^2^ = 0.94), so only starch content was used for subsequent analyses. Starch content was negatively correlated with perceived moisture in the mass (R^2^ = 0.64) and positively correlated with mealiness (R^2^ = 0.68). The gelatinization temperature and ratio of B-type starch (peak 1) were negatively correlated with sensory perception of particle size (R^2^ = 0.58) and mealiness (R^2^ = 0.55), respectively (Tables 1 and A.11). Overall, only 3 baked sweetpotato textures could potentially be predicted by univariate models, which is not surprising given the complex matrix of the sweetpotato and the enzymatic and thermally induced changes during cooking. Therefore, multivariate models were explored for further elucidation of the combined effects of multiple physicochemical properties.

**Fig. 2:**
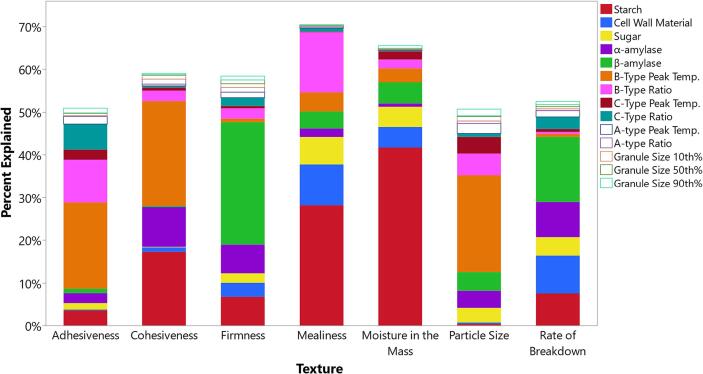
Percentage of sensory texture variance explained by raw sweetpotato attributes in random forest models with K-fold cross validations. Percent contribution of a factor to the model is represented by its portion of the column. Strong predictors are filled in, and only models with R^2^ > 0.50 are reported.

Multivariate random forests modeling was used to investigate the complex relationships between raw sweetpotato properties and baked sweetpotato textures. Random forest models accounted for more of the variance than the linear regressions, where at least half of the variance (R^2^ > 50 %) in sensory firmness, moisture in mass, mealiness, adhesiveness, cohesiveness, particle size, and rate of breakdown was explained by the multivariate models (Fig. 2, Tables 1 and A.12). Dominant predictors of baked sweetpotato texture were starch content, B-type starch gelatinization temperature and ratios, β and α-amylase activities, and cell wall material contents (Fig. 2 and Table A.12). Interestingly, linear relationships between amylase activities and textures were weak in baked sweetpotatoes (Table A.11) and sweetpotato fries (Sato et al., 2018), but amylases were significant factors in multivariate texture models for both baked (Fig. 2) and fried sweetpotato textures (Allan et al., 2023). Starch granule sizes contributed little to these texture prediction models, suggesting this starch property has little to no impact on baked sweetpotato textures. These multivariate models support part A of the hypothesis because starch thermal properties and amylase activities contributed significantly to the predictions of baked sweetpotato textures.

Sensory moisture in mass, mealiness, adhesiveness, cohesiveness, and particle size textures were better predicted by multivariate models using raw sweetpotato properties than the models with TPA parameters; however, fracturability, firmness, and rate of breakdown were better predicted by TPA. Therefore, a multiangled approach using both physical and chemical analyses may be useful for comprehensive screening of sweetpotato genotypes for texture attributes.

#### 3.3.2 Texture and composition relationships

Baked sweetpotato firmness, cohesiveness, and moisture in mass were selected for further investigation because these were representative of the sensory texture clusters (Fig. 1) and scores ranged from less than 1 to greater than 6 on the 10-point sensory scale (Figure A.2). The impact of physicochemical sweetpotato properties on each of these textural attributes will be discussed further.

The best predictors for sweetpotato firmness were β-amylase activity, starch content, and α-amylase accounting for 50.1, 11.8, and 11.7 % of the model, respectively (Fig. 2 and Table A.12). Genotypes with the highest firmness scores were NCDM04-001, NC16-0613, NCP16-0095, and Dimbuka Bukalula, while the lowest were Beauregard, Covington, and NC15-0633. Dry matter and/or starch content contributes to the material structure and have been positively correlated with cooked sweetpotato firmness (Kitahara et al., 2017, Yoon et al., 2018). However, the multivariate model suggests baked sweetpotato firmness is related to starch content but also amylase activities, which alter the starch structure during cooking. β-amylase activity was the strongest predictor of cooked sweetpotato firmness, and the most firm genotypes tended to have lower activities (Table A.9). Banda et al. (2021) also reported a negative correlation with cooked sweetpotato firmness and β-amylase activity. β-amylase converts starch to maltose and higher activities would favor greater starch breakdown and maltose production, which maltose contributes to the volume of the aqueous fraction. The α-amylase activity was also a significant factor and is an endoenzyme that breaks starch into smaller molecular weights (i.e., maltodextrins). Higher activities would suggest greater starch breakdown and thus structural loss. However, starch contents and amylase activities are not the only factors affecting sweetpotato firmness. The texture of NC16-0613 was exceptionally firm but based on the β-amylase activities (146.4 β-U), α-amylase activities (35.6 α-U), and starch contents (10.3 %), it would be expected to be much softer. Cell wall polymers also play a role in cooked sweetpotato texture (Buescher and Balmoori, 1982, Kitahara et al., 2017, Walter et al., 2003), so it is postulated that this firm genotype has unique cell wall components that resisted thermal degradation during cooking. Therefore, sweetpotato firmness is a function of multiple components and biochemical factors. More research is needed to elucidate the complex relationships with cell wall polymers. TPA could be utilized for such studies since it was highly correlated with sensory firmness (Table 1).

Genotypes with the highest cohesiveness were NCP16-0046, NCP16-0095, and Japanese, whereas the lowest were NC16-0613, NC15-0633, and NC17-0331. In the multivariate model, B-type starch gelatinization temperature (40.4 % of the model), starch content (28.4 %), and α-amylase activity (15.3 %) were significant factors (Fig. 2 and Table A.12). There was a positive relationship between B-type starch peak temperature and cohesiveness, where sweetpotatoes with B-type starch that gelatinized at higher temperatures tended to be more cohesive in texture. Starch gelatinization temperature is associated with the amylopectin structure, and higher gelatinization temperatures are correlated with longer starch branch lengths (Allan et al., 2023, Noda et al., 1998). Sweetpotato starch structure also affects isolated starch rheological properties (Zhu & Xie, 2018). Thus, the association with B-type starch peak temperature may be a result of longer amylopectin branch lengths impacting the perceived cohesiveness. Similarly, there was a negative relationship between cohesiveness and α-amylase activities. The genotypes with lower α-amylase activities would be expected to have more intact starch molecules, which may maintain more cohesive textures. Sweetpotatoes with more starch also tended to have more cohesive textures, likely by the starch increasing the local viscosity. Overall, baked sweetpotato cohesiveness was mainly influenced by the starch – both its content and properties.

The sweetpotato genotype with the lowest moisture in mass texture was NCDM04-001 and the highest were Covington, Beauregard, NC15-0633, NC17-0331, and NCP13-0315. Starch content was the strongest and only significant predictor (63.6 % of the model), which suggests other physicochemical properties contributed minimally to the prediction of moisture in mass. However, other physicochemical properties affected moisture in mass, because the multivariate model improved upon the univariate model (Table 1). The cell wall material content, sugar content, and β-amylase were not considered significant by the > 10 % criteria but combined explained a notable portion of the variance (Fig. 2). Similarly, Laurie et al. (2013) also reported the “wateriness” of sweetpotatoes was negatively correlated with dry matter. Starch imbibes large quantities of water during gelatinization and increases the viscosity (Huber & BeMiller, 2017), and both the internalization of water and increased water-starch interactions would likely affect the perceived moisture in mass. In addition, sweetpotatoes with higher starch contents have lower moisture contents. Therefore, higher starch contents decrease the perception of moisture.

### 3.4 Sweetness perception

Sugar concentrations in baked roots were measured to investigate the impacts on perceived sweetness. Glucose and fructose contents were highly correlated (r = 0.97, Table A.10) and ranged from 2.1 to 25.9 mg/g and 1.6 to 15.6 mg/g, respectively. These monosaccharide concentrations were exceptionally high in baked NC17-0331 at 25.9 and 15.6 mg/g, while the next highest concentrations were in NCMC16-0298 and O’Henry at 14.4 and 9.7 mg/g and 12.4 and 9.2 mg/g, respectively. Dimbuka Bukalula and NCDM04-001 were low in glucose and fructose with less than 2.5 mg/g (Table A.13). Sucrose contents were higher than the monosaccharides and ranged from 57.3 to 22.2 mg/g with the highest contents in NC13-1027, Covington, and NC15-0633 and the lowest in O’Henry and NCMC16-0298 (Table A.13). The high fructose and glucose concentrations and low sucrose concentrations in O’Henry and NCMC16-0298 are likely related because glucose and fructose concentrations have been inversely associated with sucrose during storage (Sakamoto, Masuda, Nishimura, & Ikeshita, 2014). Maltose concentrations in baked sweetpotatoes varied the most of the sugars, ranging from 0.8 to 108.2 mg/g (Table A.13). NCP16-0095, Japanese, and NCP16-0046 were high in maltose with more than 88 mg/g while NCDM04-001 and NC13-1027 had less than 2 mg/g of maltose due to an inactive β-amylase (<10 U/100 g) (Tables A.8). Maltose is formed mainly during cooking and is affected by starch gelatinization temperatures, β-amylase activities, and inactivation temperatures (Takahata et al., 1994). It is well known that sweetness perception of individual sugars varies; therefore, the impact of these individual sugars on perceived sweetness of baked sweetpotatoes were investigated.

Covington and Japanese genotypes were the sweetest genotypes and NCP16-0613 and NCDM04-001 were the least sweet genotypes (Table A.13). Sweetpotato sweetness scores were weakly correlated with total sugar contents in the baked roots (R^2^ = 0.34, Figure A.5 Panel A). Adjusting individual sugar contents for sucrose sweetness equivalence (sucrose 1; glucose 0.64, fructose 1.2, and maltose 0.43 (Shallenberger, 1993) improved the correlation (R^2^ = 0.60, Figure A.5 Panel B), but 40 % of variation was still unexplained. These variations in sweetness perception were hypothesized to be affected by the varying textures of the sweetpotato genotypes, as textures of foods are known to affect sweetness perception (Mosca, van de Velde, Bult, van Boekel, & Stieger, 2012).

**Fig. 3:**
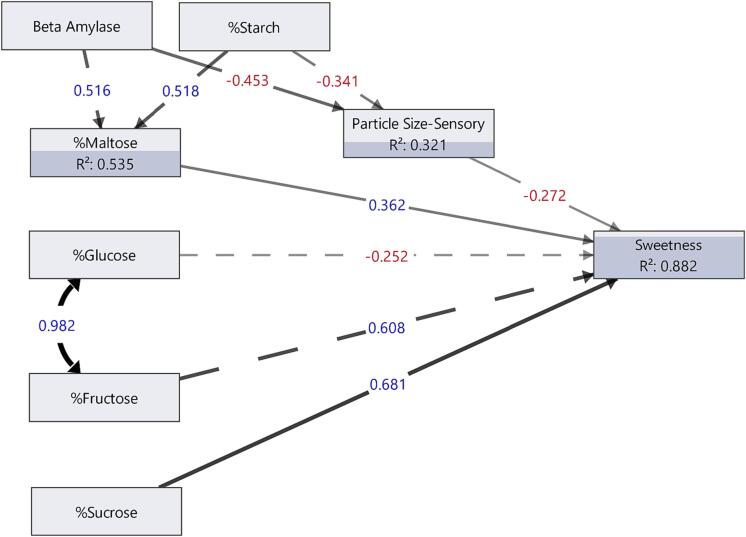
Structural equation model of sweetness perception in baked sweetpotatoes. Significant relationships are indicated by solid arrows, thickness of arrows represent coefficient magnitude, red coefficients are negative, blue coefficient are positive, and R^2^ is the percent variance explained by the inputs. (For interpretation of the references to color in this figure legend, the reader is referred to the web version of this article.)

The relationship between sugar types and textures on sweetness perception was tested using a hypothesis-based modeling approach, structural equation modeling (SEM). The following was the SEM hypothesis structure: (1) individual sugar concentrations affect sweetness perception; (2) textures affect sweetness perception; and (3) β-amylase activity and starch content affect maltose content and textures. The textures particle size, cohesiveness, mealiness, and moisture in mass were used for SEM development, but the best model based on fitting criteria included only particle size. In the SEM (Fig. 3), both sugar concentrations and perceived particle size affected sweetness perception and accounted for most of the variation (R^2^ = 0.882). Maltose and sucrose concentrations affected sweetness perception significantly, whereas glucose and fructose were not significant sweetness predictors. Although fructose is much sweeter than maltose on a per gram basis, maltose concentration was significantly higher in concentration and varied more than fructose in these genotypes (Table A.13). The perceived particle size was negatively associated with sweetness, where sweetpotatoes with smaller perceived particle sizes tended to be sweeter. Similarly, sucrose gels that broke down into smaller pieces were perceived as more sweet (Mosca et al., 2012). This model also suggests that starch content and β-amylase activity impact sweetness indirectly. For example, higher starch content and β-amylase activity would result in more maltose and somewhat lower perceived particle size, which would both positively impact sweetness. Therefore, the perceived sweetness of sweetpotato depends upon both texture and sugar concentrations.

#### 3.4.1 Maltose production

Maltose was the predominant sugar in most of the baked sweetpotatoes (Table A. 12) and a significant factor in perceived sweetness (Fig. 3); however, maltose content in baked sweetpotatoes was not individually correlated with α-, β-amylase, nor the B-type starch gelatinization temperature (R^2^ < 0.30), which are all necessary for maltose production. Interestingly, maltose production was highly correlated with starch content when β-amylase null genotypes NCDM04-001 and NC13-1027 were excluded (R^2^ = 0.90, Figure A.4). This suggests that the limiting factor for maltose production in baked sweetpotatoes is starch content, assuming there is an active β-amylase (≫10 β-amylase U/100 g; Table A.9). Takahata et al. (1994), reported a similar finding in baked sweetpotato pieces where maltose production was positively correlated with dry matter, but they proposed starch gelatinization properties and varying enzymatic activities and thermal stabilities may also affect maltose production. A random forest model of maltose production using physicochemical sweetpotato properties did not improve upon the univariate relationship between starch and maltose. Therefore, maltose content in baked sweetpotatoes, a slow cooking method, is mainly dependent on starch content contingent on some β-amylase activity.

## 4 Conclusions

Baked sweetpotato textures were predictable by either TPA or raw sweetpotato properties, each with its own limitations. The first bite and compression textures were best predicted by TPA, but TPA was not a good predictor for mouthfeel textures. These mouthfeel textures were impacted by multiple raw sweetpotato factors, most notably starch gelatinization properties, amylase activities, starch content, and total sugar contents. Our hypothesis that starch thermal properties and amylases affect textures was partially correct since this was true for some but not all textures. The perceived sweetness of baked sweetpotatoes was affected by sugar contents as well as texture, confirming our second hypothesis. Maltose content was a significant factor for sweetness in baked sweetpotatoes, which was mainly dictated by starch content along with functional amylases. Therefore, predicting sweetness perception solely on raw sugar contents provides a limited perspective, since maltose development during cooking and cooked texture greatly impact sweetness.

This work demonstrated that baked sweetpotato textures can be predicted from raw sweetpotato properties and TPA of baked roots, and sweetness perception was affected by individual sugar concentrations and texture. This study also provided a better understanding of the relationship between raw sweetpotato properties and the eating experience, which will help food scientists, sweetpotato breeders, and processors develop sweetpotato products that meet consumer preferences.

## 5 Note

Mention of trade names or commercial products in this publication is solely for the purpose of providing specific information and does not imply recommendation or endorsement by the U.S. Department of Agriculture. USDA is an equal opportunity provider and employer.

## Ethical statement

Descriptive sensory analysis was conducted in accordance with the guidance for ethical treatment of human subjects. All panelists were adult volunteers whom were not coerced nor compensated for their participation. Participants were informed about the study, the freedom to stop their participation at any time, and the use of the data for publication. Informed consent was provided by panelists at the beginning of panel training. Panelist anonymity was maintained by the use of Compusense sensory analysis software to assign panelist codes, and the data presented is not associated with any individual panelist. Food samples were prepared by individuals trained and certified in the safe handling of food.

## Funding

This research was funded by the 10.13039/100007917U.S. Department of Agriculture, Agricultural Research Service, with partial support from the SweetGAINS project paid for by the 10.13039/100000865Bill and Melinda Gates Foundation and the 10.13039/100016774International Potato Center (CIP) (Contract ID: OPP1213329). Mention of trade names or commercial products in this publication is solely for the purpose of providing specific information and does not imply recommendation or endorsement by the 10.13039/100000199U.S. Department of Agriculture. USDA is an equal opportunity provider and employer.

## CRediT authorship contribution statement

**Matthew C. Allan:** Conceptualization, Data curation, Formal analysis, Investigation, Methodology, Visualization, Writing – original draft. **Suzanne D. Johanningsmeier:** Conceptualization, Formal analysis, Funding acquisition, Methodology, Project administration, Supervision, Writing – review & editing. **Mariam Nakitto:** Investigation, Methodology, Writing – review & editing. **Osvalda Guambe:** Investigation, Methodology, Writing – review & editing. **Modesta Abugu:** Investigation, Methodology, Writing – review & editing. **Kenneth V. Pecota:** Methodology, Resources, Writing – review & editing. **G. Craig Yencho:** Funding acquisition, Methodology, Resources, Writing – review & editing, Supervision.

## Declaration of competing interest

The authors declare that they have no known competing financial interests or personal relationships that could have appeared to influence the work reported in this paper.
