# Supplementary material for: Baked sweetpotato textures and sweetness: An investigation into relationships between physicochemical and cooked attributes
Source: Food Chem X. 2023 Dec 15;21:101072. doi: 10.1016/j.fochx.2023.101072 (PMC10776778; doi:10.1016/j.fochx.2023.101072)
Supplement: Supplementary Data 1 [file mmc1.docx]

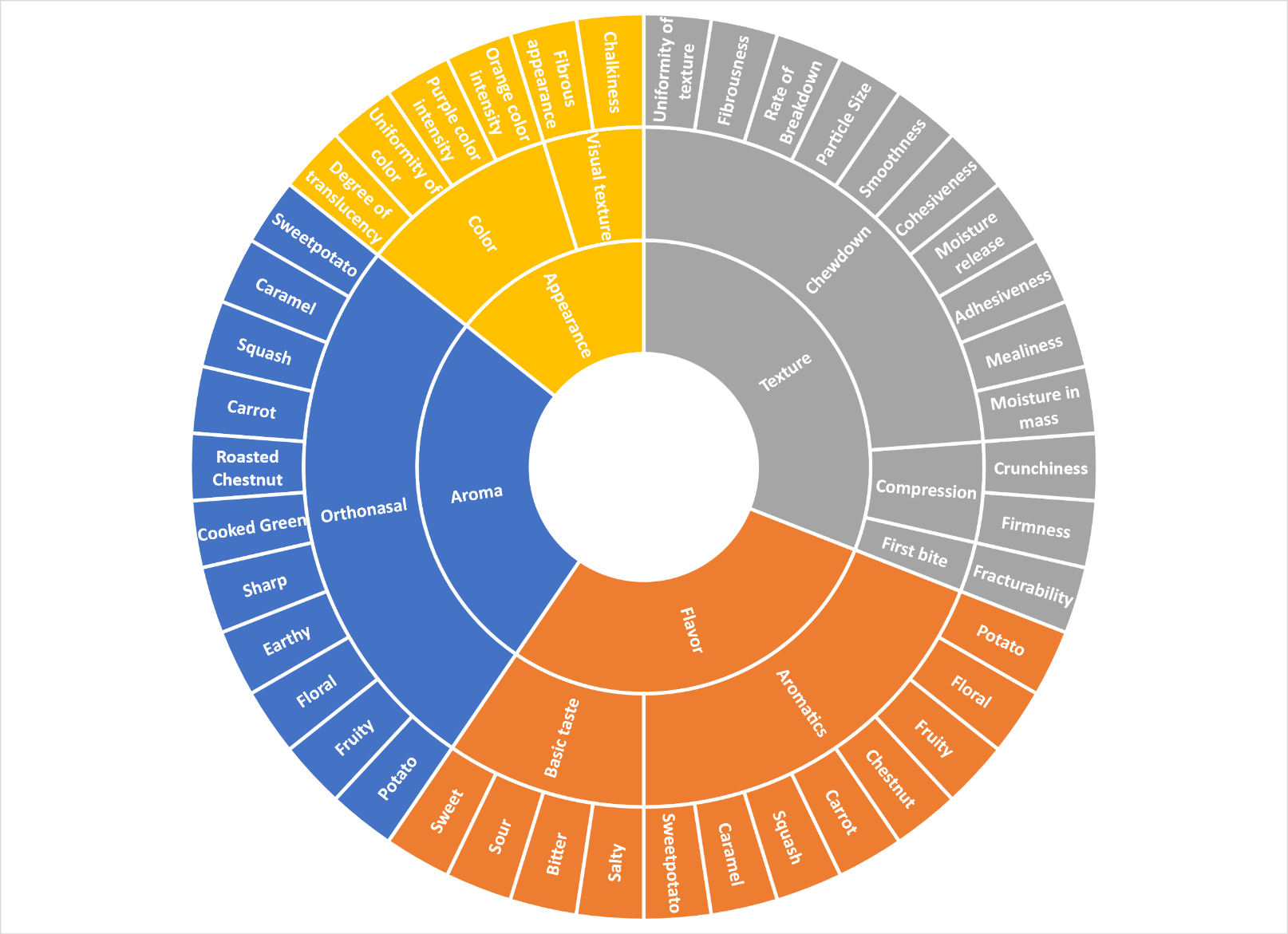
Appendix

**Sweetpotato Sensory Lexicon**

0

1

1

1

0

0

0

3

2

5

6

0

0

0

2

3

3

5

0

1

0

6

0

2

5

0

6

0

2

0

2

7

Varies

5

0

0

2

7

7

1

8

5

Figure A.1. Sweetpotato sensory lexicon wheel. Numbers on the outside of the wheel represent the scores established for the baked Covington sweetpotato reference samples.

Table A.1. Descriptions of sensory textures

| **DESCRIPTOR** | **DEFINITION** | **SCALE RANGE AND MAIN ANCHORS** | **QUALITATIVE REFERENCE** | **COVINGTON SCORE** |
| --- | --- | --- | --- | --- |
| **Taste** |  |  |  |  |
| Sweet | Taste of sucrose solution | 0: not at all sweet; 5: moderately sweet; 10: extremely sweet | 1% sucrose solution(w/v)=1; 4% sucrose solution(w/v)=4; 7% sucrose solution(w/v)=7; 10% sucrose solution(w/v)=10 | 6 |
| **Texture in mouth** |  |  |  |  |
| Fracturability | Ease with which sample is broken into distinct pieces when bitten between incisors | 0: easily deforms; 5: partly fractures; 10: easily fractures | Jicama slices (1/2” thickness) | 5 |
| Firmness in mouth | Amount of force required to compress product between molars | 0: extremely soft; 5: firm; 10: hard | Low moisture mozzarella cheese stick=firm | 2 |
| Crunchiness | Production of low-pitched sound while chewing certain foods such as cooked carrot, cooked cucumber | 0: not crunchy; 10: very crunchy | Water chestnut=crunchy | 0 |
| Moisture (in the mass) | Amount of moisture present in sample mass | 0: dry ; 10: extremely moist | Egg yolk=dry; Del Monte diced pears=moist | 6 |
| Crumbliness in mouth (mealiness) | Perception of fine powdery particles upon chewing products such as egg yolk | 0: not crumbly, 10: very crumbly | Egg yolk (mealy) | 0 |
|  |  |  |  |  |
| Adhesiveness | Amount of sample that adheres to oral surfaces | 0: none, 10: very high |  | 2 |
|  |  |  |  |  |
| Fibrousness | Presence of string like structures in mouth after chewing | 0: none, 9: pineapple core; 10: Extremely fibrous | Dole canned pineapple, Freshly sliced pineapple core | 1 |
|  |  |  |  |  |
| Smoothness | Degree of absence of grainy particles in mass sample | 0: grainy, 10: very smooth | Kroger sour cream=smooth; Del Monte diced pear = grainy; | 7 |
| Particle size | The size of sample particles after 3 – 5 chews | 0: extremely fine, 10: extremely big | Musselman’s apple butter | 5 |
| Rate of breakdown | Number of chews required to masticate a sample until you can swallow it or when it starts to dissolve | 0: very slow, 5: 8 chews; 9: 4 chews; 10: 3 chews | Pepperidge Bordeaux cookie; Thomas English muffin | 8 |
| Moisture release | Attribute of food products to release moisture when pressure is applied (when chewing) such as cooked cucumber | 0: absent; 10: very high | Cucumber | 0 |
| Cohesiveness (moldability) | Degree to which the chewed sample hold together in a mass | 0: falls apart 5: moderately cohesive 10: extremely cohesive | Cheerios; Sara Lee butter pound cake | 2 |
| Uniformity of texture | Overall impression of the degree to which the texture of the sample is evenly distributed across surface | 0: not mealy, 10: extremely mealy |  | 7 |

Table A.2. Sensory texture attributes of the 15 sweetpotato genotypes

|  | Fracturability | Firmness | Moisture in the Mass | Mealiness | Adhesiveness | Cohesiveness | Smoothness | Particle Size | Rate of Breakdown | Fibrousness | Uniformity of Texture | Crunchiness | Moisture Release |
| --- | --- | --- | --- | --- | --- | --- | --- | --- | --- | --- | --- | --- | --- |
| Beauregard | 4.26 ± 0.42 | 1.19 ± 0.47 | 5.86 ± 0.7 | 0.06 ± 0.1 | 2.38 ± 0.27 | 2.23 ± 0.67 | 7.68 ± 0.17 | 4.61 ± 0.42 | 8.71 ± 0.55 | 1.37 ± 0.34 | 7.61 ± 0.59 | 0 ± 0 | 0.47 ± 0.12 |
| Bonita | 4.99 ± 1.61 | 3.14 ± 0.76 | 3.86 ± 0.76 | 1.63 ± 0.44 | 3.02 ± 0.31 | 3.98 ± 0.77 | 7.26 ± 0.35 | 3.6 ± 0.15 | 7.24 ± 0.68 | 0.48 ± 0.5 | 8.13 ± 0.3 | 0.05 ± 0.08 | 0.15 ± 0.01 |
| Covington | 5.26 ± 0.83 | 2.15 ± 0.99 | 5.95 ± 0.5 | 0.05 ± 0.08 | 2.13 ± 0.39 | 2.15 ± 0.29 | 7.26 ± 0.48 | 4.99 ± 0.38 | 8.28 ± 0.5 | 1.59 ± 0.48 | 6.51 ± 1.07 | 0.19 ± 0.22 | 0.52 ± 0.35 |
| Dimbuka Bukalula | 6.9 ± 0.16 | 4.71 ± 0.25 | 2.79 ± 0.76 | 2.38 ± 0.44 | 2.51 ± 0.2 | 2.98 ± 1.31 | 4.63 ± 1.26 | 5.42 ± 1.42 | 5.1 ± 0.5 | 0.68 ± 0.78 | 6.18 ± 0.47 | 0.37 ± 0.27 | 0.05 ± 0.08 |
| Japanese (KokEI No. 14) | 5.86 ± 0.29 | 3.67 ± 0.08 | 3.19 ± 0.3 | 2.43 ± 0.8 | 3.14 ± 0.38 | 4.86 ± 1.13 | 7 ± 0.65 | 3.48 ± 1.45 | 5.76 ± 0.54 | 0.19 ± 0.22 | 8.33 ± 0.58 | 0 ± 0 | 0.05 ± 0.08 |
| NCMC16-0298 | 5.05 ± 1.66 | 2.71 ± 1.27 | 4.19 ± 1.03 | 2 ± 0.38 | 2.9 ± 0.08 | 3.62 ± 0.5 | 6.95 ± 0.59 | 2.67 ± 0.3 | 7.29 ± 0.8 | 0.57 ± 0.43 | 8.38 ± 0.5 | 0 ± 0 | 0.24 ± 0.16 |
| NC13-1027 | 5.29 ± 0.87 | 3.24 ± 0.87 | 4.38 ± 1.15 | 0.9 ± 0.95 | 2.05 ± 0.36 | 2.9 ± 0.79 | 5.57 ± 0.49 | 5.62 ± 0.3 | 6.9 ± 1.72 | 1.24 ± 0.64 | 6.29 ± 0.49 | 0.14 ± 0.14 | 0.29 ± 0.25 |
| NC15-0633 | 4.81 ± 1.08 | 1.67 ± 0.5 | 6.67 ± 0.36 | 0.1 ± 0.16 | 2.14 ± 0.25 | 1.62 ± 0.46 | 6.95 ± 0.36 | 5.29 ± 0.38 | 8.62 ± 0.73 | 1.52 ± 0.33 | 6.81 ± 0.58 | 0.19 ± 0.22 | 0.76 ± 0.08 |
| NC16-0613 | 8.42 ± 0.42 | 7.11 ± 1.24 | 3.46 ± 0.19 | 0.31 ± 0.18 | 0.53 ± 0.35 | 1.28 ± 1.27 | 3.05 ± 1.15 | 8.1 ± 0.5 | 2.99 ± 0.66 | 0.3 ± 0.15 | 6.25 ± 0.36 | 3.64 ± 2.06 | 1.27 ± 0.86 |
| NC17-0331 | 5.28 ± 1.32 | 2.47 ± 1.42 | 6.09 ± 0.78 | 0.14 ± 0.25 | 1.86 ± 0.25 | 1.68 ± 0.42 | 6.15 ± 1.09 | 5.66 ± 0.87 | 7.81 ± 1.21 | 1.75 ± 0.16 | 6.45 ± 1.2 | 0.24 ± 0.22 | 0.49 ± 0.32 |
| NCDM04-001 | 8.42 ± 0.74 | 6.84 ± 0.26 | 0.4 ± 0.11 | 4.44 ± 0.73 | 2.56 ± 0.51 | 2.05 ± 0.5 | 2.75 ± 0.07 | 5.6 ± 0.51 | 2.98 ± 0.63 | 0.64 ± 0.19 | 5.96 ± 0.18 | 0.24 ± 0.3 | 0 ± 0 |
| NCP13-0315 | 4.79 ± 0.91 | 2.27 ± 0.51 | 5.9 ± 0.58 | 0.37 ± 0.34 | 2.91 ± 0.62 | 2.63 ± 0.11 | 6.67 ± 0.45 | 4.75 ± 0.85 | 7.43 ± 1.11 | 2.77 ± 0.53 | 6.68 ± 0.83 | 0.05 ± 0.08 | 0.55 ± 0.43 |
| NCP16-0046 | 5.05 ± 0.86 | 3.71 ± 0.76 | 3.48 ± 0.22 | 2 ± 0.25 | 3.52 ± 0.46 | 5.05 ± 0.16 | 6.52 ± 0.72 | 3.19 ± 0.5 | 5.67 ± 0.54 | 0.76 ± 0.44 | 8.1 ± 0.79 | 0 ± 0 | 0.1 ± 0.08 |
| NCP16-0095 | 7.67 ± 0.97 | 4.76 ± 0.08 | 2.71 ± 0.43 | 1.71 ± 0.29 | 2.86 ± 0.62 | 5 ± 1.22 | 5.05 ± 1.3 | 4.57 ± 1.29 | 4.48 ± 0.7 | 1.67 ± 0.97 | 6.38 ± 0.7 | 0.1 ± 0.16 | 0.05 ± 0.08 |
| O'Henry | 4.57 ± 1 | 2.57 ± 0.62 | 4.1 ± 0.58 | 2.1 ± 0.36 | 2.95 ± 0.36 | 3.43 ± 0.29 | 6.9 ± 0.46 | 3.14 ± 0.76 | 7.38 ± 0.79 | 0.52 ± 0.41 | 8.19 ± 0.5 | 0 ± 0 | 0.29 ± 0.14 |


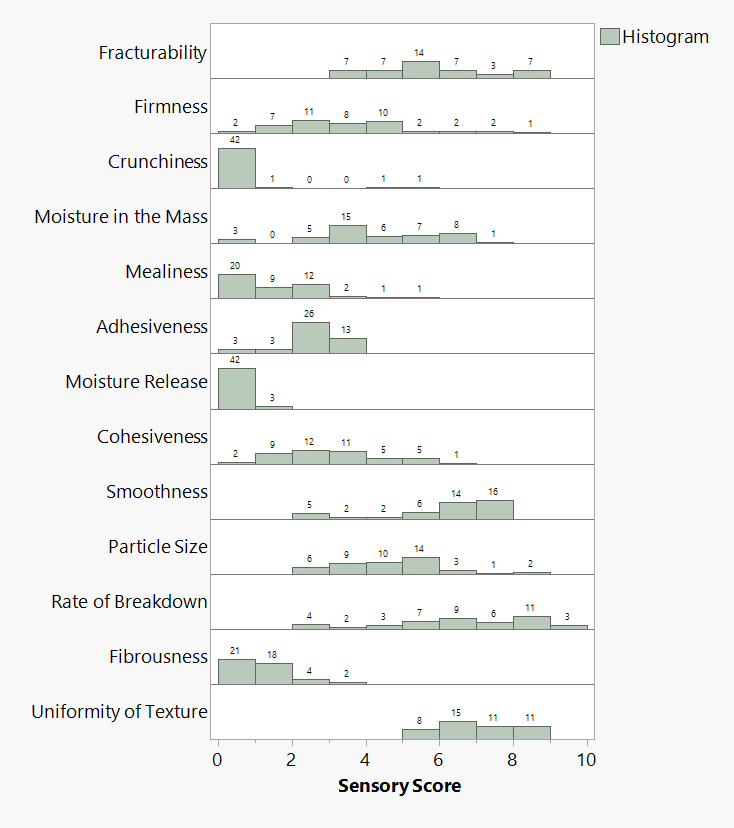


Figure A.2. Distributions of baked sweetpotato sensory textures

Table A.3. Principle component (Figure 1 in manuscript) loading matrix.

| **Texture** | **Prin1** | **Prin2** | **Prin3** | **Prin4** | **Prin5** | **Prin6** | **Prin7** | **Prin8** | **Prin9** | **Prin10** | **Prin11** |
| --- | --- | --- | --- | --- | --- | --- | --- | --- | --- | --- | --- |
| Fracturability | -0.92513 | -0.01026 | 0.03826 | 0.22207 | 0.19516 | -0.17556 | 0.02985 | 0.03478 | 0.12434 | 0.08054 | -0.01723 |
| Firmness | -0.96346 | 0.10929 | -0.02614 | 0.08722 | 0.09551 | 0.10108 | 0.08166 | -0.03380 | 0.00775 | -0.08957 | 0.12757 |
| Moisture in the Mass | 0.77702 | -0.58151 | -0.05769 | 0.04856 | -0.06289 | -0.01499 | 0.08283 | -0.15272 | 0.03863 | 0.10718 | 0.07107 |
| Mealiness | -0.49010 | 0.74870 | 0.08558 | -0.39796 | 0.01305 | -0.06197 | -0.11962 | 0.00309 | -0.04529 | 0.09532 | 0.06398 |
| Adhesiveness | 0.35387 | 0.77793 | 0.40620 | -0.15975 | -0.10098 | -0.02557 | 0.24518 | 0.02654 | 0.08439 | -0.01537 | -0.00756 |
| Cohesiveness | 0.09249 | 0.81737 | 0.24999 | 0.45375 | -0.19381 | 0.00508 | -0.12746 | -0.00697 | 0.00122 | 0.01768 | 0.02761 |
| Smoothness | 0.92474 | 0.16935 | -0.04881 | 0.15410 | 0.14032 | -0.17892 | 0.09244 | 0.08859 | -0.14045 | 0.00175 | 0.04709 |
| Particle Size | -0.58888 | -0.74875 | -0.03744 | 0.00759 | -0.22941 | 0.03242 | 0.06195 | 0.16995 | -0.01476 | 0.05523 | 0.03824 |
| Rate of Breakdown | 0.92977 | -0.23217 | -0.04526 | -0.11066 | -0.00311 | -0.10057 | -0.14717 | 0.07632 | 0.14612 | -0.06735 | 0.06189 |
| Fibrousness | 0.34293 | -0.47340 | 0.77276 | 0.01349 | 0.16633 | 0.16684 | -0.05201 | 0.03905 | -0.01242 | 0.03163 | 0.00866 |
| Uniformity of Texture | 0.57682 | 0.59704 | -0.46758 | 0.05460 | 0.10833 | 0.25254 | 0.02430 | 0.07777 | 0.05182 | 0.06611 | 0.00294 |

Table A.4. Correlation coefficients (r) between sensory texture attributes.

|  | Fracturability | Firmness | Crunchiness | Moisture in the Mass | Mealiness | Adhesiveness | Moisture Release | Cohesiveness | Smoothness | Particle Size | Rate of Breakdown | Fibrousness | Uniformity of Texture |
| --- | --- | --- | --- | --- | --- | --- | --- | --- | --- | --- | --- | --- | --- |
| Fracturability | 1 | 0.9023 | 0.4882 | -0.7046 | 0.3715 | -0.3529 | 0.0264 | -0.0253 | -0.7784 | 0.512 | -0.8571 | -0.276 | -0.5536 |
| Firmness | 0.9023 | 1 | 0.5911 | -0.8023 | 0.5015 | -0.2725 | 0.0228 | 0.007 | -0.8531 | 0.4677 | -0.9396 | -0.3758 | -0.4434 |
| Crunchiness | 0.4882 | 0.5911 | 1 | -0.116 | -0.1921 | -0.6783 | 0.6899 | -0.422 | -0.5836 | 0.6531 | -0.4876 | -0.2329 | -0.3076 |
| Moisture in the Mass | -0.7046 | -0.8023 | -0.116 | 1 | -0.8377 | -0.1845 | 0.4362 | -0.3893 | 0.6165 | -0.0185 | 0.8354 | 0.4781 | 0.1195 |
| Mealiness | 0.3715 | 0.5015 | -0.1921 | -0.8377 | 1 | 0.4726 | -0.5736 | 0.4232 | -0.3802 | -0.2817 | -0.5744 | -0.4594 | 0.0898 |
| Adhesiveness | -0.3529 | -0.2725 | -0.6783 | -0.1845 | 0.4726 | 1 | -0.6429 | 0.6853 | 0.4177 | -0.7676 | 0.1294 | 0.0304 | 0.4639 |
| Moisture Release | 0.0264 | 0.0228 | 0.6899 | 0.4362 | -0.5736 | -0.6429 | 1 | -0.6277 | -0.1263 | 0.5078 | 0.1015 | 0.1358 | -0.1933 |
| Cohesiveness | -0.0253 | 0.007 | -0.422 | -0.3893 | 0.4232 | 0.6853 | -0.6277 | 1 | 0.2423 | -0.6348 | -0.1463 | -0.1802 | 0.4272 |
| Smoothness | -0.7784 | -0.8531 | -0.5836 | 0.6165 | -0.3802 | 0.4177 | -0.1263 | 0.2423 | 1 | -0.6816 | 0.7986 | 0.1957 | 0.6379 |
| Particle Size | 0.512 | 0.4677 | 0.6531 | -0.0185 | -0.2817 | -0.7676 | 0.5078 | -0.6348 | -0.6816 | 1 | -0.375 | 0.0966 | -0.7677 |
| Rate of Breakdown | -0.8571 | -0.9396 | -0.4876 | 0.8354 | -0.5744 | 0.1294 | 0.1015 | -0.1463 | 0.7986 | -0.375 | 1 | 0.3822 | 0.3927 |
| Fibrousness | -0.276 | -0.3758 | -0.2329 | 0.4781 | -0.4594 | 0.0304 | 0.1358 | -0.1802 | 0.1957 | 0.0966 | 0.3822 | 1 | -0.382 |
| Uniformity of Texture | -0.5536 | -0.4434 | -0.3076 | 0.1195 | 0.0898 | 0.4639 | -0.1933 | 0.4272 | 0.6379 | -0.7677 | 0.3927 | -0.382 | 1 |

Table A.5.Texture profile analysis of 15 baked sweetpotato genotypes

|  | Hardness | Fracturability | Adhesiveness | Springiness | Cohesiveness | Gumminess | Chewiness | Resilience |
| --- | --- | --- | --- | --- | --- | --- | --- | --- |
| Beauregard | 361.25 ± 37.69 | 310.24 ± 51.95 | -18.83 ± 7.89 | 0.22 ± 0.03 | 0.13 ± 0.00 | 45.44 ± 4.82 | 10.34 ± 1.43 | 0.05 ± 0.01 |
| Bonita | 629.88 ± 44.61 | 501.06 ± 66.92 | -33.39 ± 1.67 | 0.24 ± 0.01 | 0.12 ± 0.01 | 71.13 ± 8.75 | 16.93 ± 1.74 | 0.05 ± 0.01 |
| Covington | 494.73 ± 180.29 | 549.41 ± 303.85 | -19.38 ± 5.17 | 0.19 ± 0.00 | 0.12 ± 0.01 | 57.14 ± 11.5 | 10.87 ± 1.93 | 0.05 ± 0 |
| Dimbuka Bukalula | 1575.06 ± 153.55 | 1854.11 ± 565.77 | -30.75 ± 8.34 | 0.21 ± 0.02 | 0.09 ± 0.02 | 145.37 ± 32.5 | 31.07 ± 6.21 | 0.05 ± 0.01 |
| Japanese (Kokei No. 14) | 1020.02 ± 61.26 | 815.42 ± 41.37 | -31.58 ± 14.52 | 0.22 ± 0.03 | 0.11 ± 0.01 | 110 ± 18.22 | 25.2 ± 5.57 | 0.05 ± 0 |
| NCMC16-0298 | 618.79 ± 106.23 | 447.75 ± 108.61 | -32.1 ± 0.87 | 0.26 ± 0.03 | 0.13 ± 0.01 | 79.36 ± 8.48 | 20.25 ± 0.35 | 0.05 ± 0.01 |
| NC13-1027 | 520.18 ± 69.05 | 455.29 ± 49.64 | -21.22 ± 2.0 | 0.21 ± 0.03 | 0.1 ± 0.01 | 54.47 ± 10.79 | 11.44 ± 3.13 | 0.05 ± 0.01 |
| NC15-0633 | 354.79 ± 77.0 | 276.84 ± 112.07 | -13.18 ± 2.94 | 0.18 ± 0.02 | 0.11 ± 0.00 | 38.98 ± 8.69 | 6.78 ± 1.05 | 0.05 ± 0.01 |
| NC16-0613 | 4231.07 ± 2491.93 | 7807.17 ± 6209.38 | -17.55 ± 5.27 | 0.35 ± 0.07 | 0.08 ± 0.01 | 347.92 ± 180.45 | 125.33 ± 66.55 | 0.05 ± 0.01 |
| NC17-0331 | 602.58 ± 287.84 | 513.73 ± 201.24 | -17.44 ± 5.44 | 0.21 ± 0.04 | 0.11 ± 0.02 | 58.8 ± 23.57 | 12.94 ± 7.24 | 0.05 ± 0 |
| NCDM04-001 | 2789.57 ± 761.58 | 3025.69 ± 554.7 | -24.27 ± 2.78 | 0.31 ± 0.00 | 0.09 ± 0.02 | 281.15 ± 130.71 | 93.49 ± 39.4 | 0.06 ± 0.01 |
| NCP13-0315 | 415.72 ± 43.88 | 334.35 ± 15.26 | -23.52 ± 8.08 | 0.21 ± 0.01 | 0.14 ± 0.02 | 56.73 ± 10.69 | 11.99 ± 1.79 | 0.06 ± 0.01 |
| NCP16-0046 | 640.79 ± 219.85 | 487.79 ± 64.34 | -34.21 ± 5.18 | 0.27 ± 0.03 | 0.13 ± 0.00 | 83.5 ± 27.15 | 22.51 ± 5.97 | 0.05 ± 0.01 |
| NCP16-0095 | 1754.46 ± 189.19 | 2019.11 ± 176.27 | -37.61 ± 3.28 | 0.29 ± 0.04 | 0.09 ± 0.00 | 167.7 ± 21.73 | 47.21 ± 5.48 | 0.05 ± 0 |
| O'Henry | 510.95 ± 60.91 | 446.26 ± 85.25 | -36.07 ± 7.45 | 0.25 ± 0.05 | 0.14 ± 0.02 | 68.3 ± 4.76 | 17.3 ± 3.44 | 0.05 ± 0.01 |

Table A.6. Correlation coefficients (r) between texture profile analysis textures.

|  | Log(Hardness) (g) | Fracturability (g) | Adhesiveness (g*sec) | Springiness (%) | Cohesiveness (%) | Log(Gumminess) | Log(Chewiness) | Resilience (%) |
| --- | --- | --- | --- | --- | --- | --- | --- | --- |
| Log(Hardness) (g) | 1 | 0.7636 | -0.1497 | 0.6587 | -0.6974 | 0.9799 | 0.9532 | 0.0577 |
| Fracturability (g) | 0.7636 | 1 | 0.1792 | 0.5154 | -0.5681 | 0.7379 | 0.721 | -0.0037 |
| Adhesiveness (g*sec) | -0.1497 | 0.1792 | 1 | -0.2955 | -0.1238 | -0.2025 | -0.2531 | 0.3302 |
| Springiness (%) | 0.6587 | 0.5154 | -0.2955 | 1 | -0.2429 | 0.7064 | 0.8185 | -0.1132 |
| Cohesiveness (%) | -0.6974 | -0.5681 | -0.1238 | -0.2429 | 1 | -0.5425 | -0.4916 | 0.3365 |
| Log(Gumminess) | 0.9799 | 0.7379 | -0.2025 | 0.7064 | -0.5425 | 1 | 0.9826 | 0.162 |
| Log(Chewiness) | 0.9532 | 0.721 | -0.2531 | 0.8185 | -0.4916 | 0.9826 | 1 | 0.0923 |
| Resilience (%) | 0.0577 | -0.0037 | 0.3302 | -0.1132 | 0.3365 | 0.162 | 0.0923 | 1 |

Table A.7. Correlations of sensory texture attributes to texture profile analysis parameters. Emboldened were the strongest significant correlations with the positive/negative relationship in parentheses and the R^2^ of a K-fold (K = 5) cross validation.

| Linear Correlations (R^2^) | | | | | |
| --- | --- | --- | --- | --- | --- |
|  | Log (Hardness) | Springiness | Cohesiveness | Log (Gumminess) | Log (Chewiness) |
| Fracturability | **0.735 (+, 0.681)** | 0.333 | 0.478 | 0.662 | 0.638 |
| Firmness | **0.885 (+, 0.860)** | 0.459 | 0.447 | 0.842 | 0.830 |
| Moisture in the Mass | 0.561 | 0.376 | 0.162 | 0.594 | **0.628 (-, 0.246)** |
| Mealiness | 0.225 | 0.139 | 0.036 | 0.258 | 0.281 |
| Adhesiveness | 0.092 | 0.016 | 0.218 | 0.052 | 0.037 |
| Cohesiveness | 0.000 | 0.015 | 0.040 | 0.002 | 0.005 |
| Smoothness | **0.665 (-, 0.584)** | 0.410 | 0.484 | 0.582 | 0.597 |
| Particle Size | 0.196 | 0.053 | 0.429 | 0.113 | 0.095 |
| Rate of Breakdown | 0.814 | 0.529 | 0.326 | 0.812 | **0.832 (-, 0.796)** |
| Fibrousness | 0.153 | 0.159 | 0.045 | 0.159 | 0.190 |
| Uniformity of Texture | 0.144 | 0.017 | 0.277 | 0.088 | 0.065 |


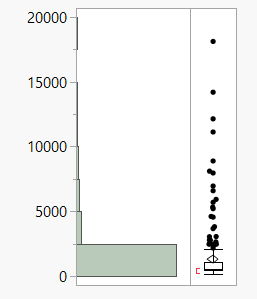

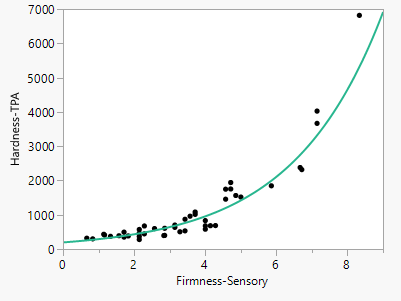


Figure A.3. TPA hardness in relation to sensory firmness scores (left) and distribution of TPA hardness (right).

Table A.8. Carbohydrate composition of 15 diverse sweetpotato genotypes.

|  | Dry Matter | Starch Content (Fresh Basis, Raw) | Cell Wall Material (DM-(Starch+Sugar)) (raw) | Glucose (mg/g) Raw | Fructose (mg/g) Raw | Sucrose (mg/g) raw | Maltose (mg/g) raw | Glucose (mg/g) baked | Fructose (mg/g) baked | Sucrose (mg/g) baked | Maltose (mg/g) baked |
| --- | --- | --- | --- | --- | --- | --- | --- | --- | --- | --- | --- |
| Beauregard | 21.4 ± 2.51 | 9.4 ± 0.95 | 6.19 ± 0.95 | 10.19 ± 2.5 | 8.17 ± 2.1 | 39.72 ± 11.08 | 0 ± 0 | 11.89 ± 2.76 | 8.24 ± 2.38 | 41.16 ± 12.32 | 42.94 ± 6.58 |
| Bonita | 27.04 ± 0.69 | 16 ± 0.61 | 6.48 ± 0.43 | 5.29 ± 0.2 | 5.99 ± 0.15 | 34.36 ± 2.76 | 0 ± 0 | 9.58 ± 4.62 | 7.41 ± 2.57 | 30.81 ± 14.79 | 62.76 ± 3.58 |
| Covington | 20.86 ± 0.25 | 8.53 ± 0.46 | 6.06 ± 0.34 | 8 ± 1.73 | 5.66 ± 0.85 | 49.02 ± 2.64 | 0 ± 0 | 9.74 ± 2.51 | 5.94 ± 1.17 | 52.23 ± 4.99 | 36.8 ± 3.42 |
| Dimbuka Bukalula | 30.2 ± 4.53 | 18.6 ± 4.48 | 8.31 ± 0.47 | 1.7 ± 0.32 | 1.36 ± 0.23 | 29.85 ± 2.33 | 0 ± 0 | 2.36 ± 0.37 | 1.61 ± 0.21 | 39.66 ± 3.15 | 83.41 ± 10.17 |
| Japanese (Kokei No. 14) | 34.05 ± 2.72 | 21.4 ± 2.38 | 8.97 ± 0.36 | 4.52 ± 0.97 | 3.36 ± 0.85 | 28.93 ± 1.6 | 0 ± 0 | 6.08 ± 1.36 | 4.75 ± 1.33 | 37.22 ± 1.09 | 94.46 ± 14.22 |
| NCMC16-0298 | 26.46 ± 0.6 | 16.67 ± 0.5 | 5.75 ± 0.37 | 12.02 ± 1.19 | 7.92 ± 0.66 | 20.49 ± 0.55 | 0 ± 0 | 14.40 ± 1.61 | 9.66 ± 0.97 | 22.18 ± 1.13 | 60.68 ± 3.46 |
| NC13-1027 | 25.89 ± 0.66 | 12.57 ± 1.12 | 6.75 ± 0.23 | 4.84 ± 1.17 | 3.43 ± 1.05 | 57.53 ± 4.91 | 0 ± 0 | 6.27 ± 1.2 | 3.71 ± 0.66 | 57.32 ± 2.48 | 0.79 ± 0.7 |
| NC15-0633 | 20.5 ± 0.79 | 7.5 ± 0.36 | 6.91 ± 0.56 | 4.33 ± 0.55 | 3.18 ± 0.37 | 53.47 ± 4.94 | 0 ± 0 | 6.05 ± 0.41 | 3.44 ± 0.14 | 52.08 ± 0.81 | 30.62 ± 1.39 |
| NC16-0613 | 22.22 ± 2.89 | 10.33 ± 2.66 | 6.97 ± 0.26 | 2.36 ± 0.7 | 2.29 ± 0.58 | 44.52 ± 0.85 | 0 ± 0 | 3.97 ± 0.59 | 2.57 ± 0.52 | 47.3 ± 4.65 | 46.01 ± 12.95 |
| NC17-0331 | 16.64 ± 1.01 | 4.27 ± 0.71 | 5.93 ± 0.34 | 20.82 ± 3.18 | 12.77 ± 1.76 | 30.9 ± 3.84 | 0 ± 0 | 25.92 ± 3.22 | 15.58 ± 2.51 | 33.86 ± 3.29 | 19.54 ± 6.72 |
| NCDM04-001 | 37.18 ± 4.22 | 22.27 ± 4.28 | 11.38 ± 0.73 | 1.86 ± 0.53 | 0.77 ± 0.4 | 32.66 ± 4.03 | 0 ± 0 | 2.08 ± 0.59 | 0.91 ± 0.32 | 28.63 ± 2.64 | 1.75 ± 0.69 |
| NCP13-0315 | 24.56 ± 2.07 | 11.9 ± 1.71 | 7.9 ± 0.68 | 11.17 ± 1.86 | 7.44 ± 0.91 | 29.03 ± 2.52 | 0 ± 0 | 12.23 ± 0.8 | 7.59 ± 0.95 | 31.89 ± 4.32 | 52.68 ± 8.84 |
| NCP16-0046 | 29.28 ± 1.16 | 17.53 ± 0.91 | 8.77 ± 0.42 | 5.14 ± 0.55 | 3.52 ± 0.43 | 21.11 ± 2.08 | 0 ± 0 | 5.62 ± 0.43 | 3.79 ± 0.23 | 27.57 ± 2.15 | 88.11 ± 3.33 |
| NCP16-0095 | 35.07 ± 2.03 | 23.67 ± 2.28 | 7.49 ± 0.3 | 1.87 ± 0.45 | 1.33 ± 0.42 | 35.98 ± 3.51 | 0 ± 0 | 3.09 ± 0.78 | 1.69 ± 0.59 | 36.15 ± 3.38 | 108.17 ± 10.8 |
| O'Henry | 23.03 ± 1.32 | 14.07 ± 0.85 | 5.27 ± 0.37 | 12.98 ± 0.94 | 9.84 ± 1.32 | 14.14 ± 2.88 | 0 ± 0 | 12.39 ± 4.9 | 9.22 ± 2.38 | 22.4 ± 8.48 | 61.55 ± 3.15 |

Table A.9. Amylase activities and starch properties of 15 diverse sweetpotato genotypes.

|  | Alpha Ceralpha Unit (U/100g FW) | Betamyl-3 Unit (U/100g FW) | B-type Peak Temperature (°C) | B-Type Ratio * | C-Type Peak Temperature (°C) | C-Type Ratio* | A-Type Peak Temperature (°C) | A-Type Ratio* | Granule Size 10th% (um) | Granule Size 50th% (um) | Granule Size 90th% (um) |
| --- | --- | --- | --- | --- | --- | --- | --- | --- | --- | --- | --- |
| Beauregard | 57.54 ± 53.36 | 298.95 ± 43.61 | 61.2 ± 1.19 | 0.55 ± 0.09 | 70.1 ± 0.86 | 0.24 ± 0.05 | 78.23 ± 1.32 | 0.21 ± 0.04 | 5.27 ± 0.58 | 14.2 ± 1.11 | 27.58 ± 1.9 |
| Bonita | 17.31 ± 1.09 | 292.58 ± 40.51 | 66.62 ± 1.66 | 0.17 ± 0.04 | 75.09 ± 3.33 | 0.59 ± 0.2 | 81.09 ± 3.26 | 0.25 ± 0.18 | 8.28 ± 1.33 | 15.68 ± 1.86 | 25.64 ± 2.77 |
| Covington | 77.18 ± 15.66 | 364.7 ± 15.95 | 61.35 ± 0.81 | 0.44 ± 0.19 | 70.79 ± 0.9 | 0.34 ± 0.18 | 79.2 ± 0.36 | 0.23 ± 0.02 | 5.68 ± 1.4 | 14.2 ± 2.63 | 26.48 ± 4.35 |
| Dimbuka Bukalula | 5.86 ± 3.98 | 192.73 ± 46.02 | 60.37 ± 3.39 | 0.19 ± 0.08 | 66.58 ± 5.23 | 0.6 ± 0.09 | 73.99 ± 5.88 | 0.21 ± 0.08 | 5.21 ± 1.02 | 13.61 ± 1.85 | 26.23 ± 3.7 |
| Japanese (Kokei No. 14) | 16.47 ± 4.96 | 349.35 ± 135.81 | 66.25 ± 1.62 | 0.14 ± 0.03 | 72.79 ± 2.02 | 0.65 ± 0.16 | 79.37 ± 3.14 | 0.21 ± 0.13 | 6.83 ± 0.64 | 14.69 ± 1.27 | 26.35 ± 2.98 |
| NCMC16-0298 | 15.63 ± 4.88 | 643.84 ± 286.87 | 71.48 ± 0.75 | 0.16 ± 0.01 | 77.68 ± 0.91 | 0.63 ± 0.1 | 82.9 ± 1.18 | 0.21 ± 0.09 | 7.02 ± 0.9 | 14.63 ± 1.69 | 24.84 ± 3.07 |
| NC13-1027 | 23.97 ± 5.06 | 9.24 ± 3.32 | 60.6 ± 0.57 | 0.58 ± 0.02 | 69.47 ± 0.75 | 0.24 ± 0.03 | 77.48 ± 0.6 | 0.18 ± 0.01 | 4.92 ± 0.38 | 13.37 ± 1.11 | 26.12 ± 2.09 |
| NC15-0633 | 44.21 ± 8.25 | 302.25 ± 55.14 | 62.02 ± 0.54 | 0.59 ± 0.02 | 70.74 ± 0.89 | 0.23 ± 0.02 | 78.46 ± 0.82 | 0.18 ± 0 | 6.19 ± 0.11 | 15.97 ± 0.85 | 30.25 ± 2.15 |
| NC16-0613 | 35.62 ± 8.27 | 146.43 ± 66.44 | 59.15 ± 1.87 | 0.63 ± 0.01 | 67.95 ± 1.82 | 0.18 ± 0.02 | 77.09 ± 0.84 | 0.19 ± 0.01 | 5.23 ± 0.85 | 15.57 ± 1.63 | 30.53 ± 2.93 |
| NC17-0331 | 36.74 ± 7.35 | 398.91 ± 78.52 | 61.44 ± 0.58 | 0.59 ± 0.1 | 67.93 ± 0.27 | 0.25 ± 0.04 | 74.85 ± 0.63 | 0.16 ± 0.07 | 2.94 ± 0.08 | 8.87 ± 0.21 | 17.43 ± 0.72 |
| NCDM04-001 | 36.73 ± 3.79 | 10.63 ± 0.76 | 63.82 ± 0.65 | 0.16 ± 0 | 70.72 ± 1.23 | 0.48 ± 0.2 | 76.69 ± 0.94 | 0.35 ± 0.2 | 5.98 ± 1.32 | 14.13 ± 1.36 | 25.7 ± 1.5 |
| NCP13-0315 | 16.98 ± 3.48 | 416.8 ± 96.78 | 63.82 ± 0.9 | 0.34 ± 0.04 | 71.78 ± 0.97 | 0.34 ± 0.08 | 78.98 ± 0.39 | 0.32 ± 0.12 | 6.55 ± 0.28 | 14.81 ± 0.76 | 26.31 ± 2.85 |
| NCP16-0046 | 7.36 ± 0.81 | 404.72 ± 17.17 | 66.56 ± 0.36 | 0.26 ± 0.04 | 74.82 ± 0.52 | 0.53 ± 0.02 | 83.79 ± 0.73 | 0.21 ± 0.02 | 7.31 ± 0.45 | 14.99 ± 0.54 | 26.19 ± 0.72 |
| NCP16-0095 | 5.45 ± 4.16 | 626.09 ± 114.14 | 66.3 ± 1.85 | 0.21 ± 0.06 | 72.5 ± 2.61 | 0.5 ± 0.14 | 78.47 ± 2.37 | 0.29 ± 0.09 | 7.92 ± 1.7 | 16.03 ± 1.44 | 27.43 ± 0.73 |
| O'Henry | 14.21 ± 0.83 | 271.5 ± 13.38 | 0 ± 0 | 0 ± 0 | 68.18 ± 1.1 | 0.35 ± 0.13 | 75.52 ± 1.02 | 0.65 ± 0.13 | 6.54 ± 0.24 | 12.89 ± 0.21 | 21.15 ± 0.31 |

*Starch granule type ratio is the ratio of a given starch crystal type from a deconvoluted sweetpotato starch gelatinization thermogram (e.g., area of B-type starch / [sum of B, C, and A areas]).

Table A.10. Correlation coefficients (r) among raw sweetpotato properties

|  | Dry Matter | Starch Content (Fresh Basis, Raw) | Glucose (mg/g) Raw | Fructose (mg/g) Raw | Sucrose (mg/g) raw | Cell Wall Material | Alpha Ceralpha Unit (U/100g FW) | Betamyl-3 Unit (U/100g FW) | B-type Peak | B-Type Ratio | C-Type Peak | C-Type Ratio | A-Type Peak | A-Type Ratio | Granule Size 10th% (um) | Granule Size 50th% (um) | Granule Size 90th% (um) |
| --- | --- | --- | --- | --- | --- | --- | --- | --- | --- | --- | --- | --- | --- | --- | --- | --- | --- |
| Dry Matter | 1 | 0.97 | -0.6274 | -0.6632 | -0.2212 | 0.7447 | -0.4088 | -0.0512 | 0.4198 | -0.7169 | 0.3151 | 0.6087 | 0.2006 | 0.0573 | 0.5107 | 0.3912 | 0.2353 |
| Starch Content (Fresh Basis, Raw) | 0.97 | 1 | -0.5674 | -0.5774 | -0.3718 | 0.5994 | -0.5161 | 0.0603 | 0.5207 | -0.7836 | 0.3743 | 0.6843 | 0.2424 | 0.1415 | 0.599 | 0.4054 | 0.1871 |
| Glucose (mg/g) Raw | -0.6274 | -0.5674 | 1 | 0.9692 | -0.3336 | -0.5756 | 0.1006 | 0.2966 | 0.0761 | 0.2896 | -0.0321 | -0.2262 | -0.0698 | 0.1093 | -0.3458 | -0.5567 | -0.5922 |
| Fructose (mg/g) Raw | -0.6632 | -0.5774 | 0.9692 | 1 | -0.3413 | -0.6647 | 0.1029 | 0.277 | 0.0912 | 0.281 | -0.0002 | -0.2175 | -0.0274 | 0.1617 | -0.2574 | -0.4873 | -0.556 |
| Sucrose (mg/g) raw | -0.2212 | -0.3718 | -0.3336 | -0.3413 | 1 | -0.0618 | 0.5627 | -0.3526 | -0.618 | 0.668 | -0.2598 | -0.5 | -0.1198 | -0.4186 | -0.2976 | 0.1225 | 0.4047 |
| AIS (DM-Starch-Sugar) (raw) | 0.7447 | 0.5994 | -0.5756 | -0.6647 | -0.0618 | 1 | -0.1436 | -0.3009 | 0.046 | -0.4419 | 0.0292 | 0.2999 | -0.0096 | -0.0494 | 0.134 | 0.1877 | 0.2077 |
| Alpha Ceralpha Unit (U/100g FW) | -0.4088 | -0.5161 | 0.1006 | 0.1029 | 0.5627 | -0.1436 | 1 | -0.1295 | -0.4352 | 0.525 | -0.2082 | -0.4767 | -0.0736 | -0.1621 | -0.4025 | -0.2004 | 0.0002 |
| Betamyl-3 Unit (U/100g FW) | -0.0512 | 0.0603 | 0.2966 | 0.277 | -0.3526 | -0.3009 | -0.1295 | 1 | 0.57 | -0.2346 | 0.4642 | 0.2138 | 0.3624 | -0.0051 | 0.2859 | 0.0089 | -0.2069 |
| B-type Peak | 0.4198 | 0.5207 | 0.0761 | 0.0912 | -0.618 | 0.046 | -0.4352 | 0.57 | 1 | -0.6597 | 0.915 | 0.669 | 0.7213 | 0.0269 | 0.7082 | 0.3308 | -0.0436 |
| B-Type Ratio | -0.7169 | -0.7836 | 0.2896 | 0.281 | 0.668 | -0.4419 | 0.525 | -0.2346 | -0.6597 | 1 | -0.4835 | -0.8742 | -0.2748 | -0.3143 | -0.6188 | -0.2895 | 0.0231 |
| C-Type Peak | 0.3151 | 0.3743 | -0.0321 | -0.0002 | -0.2598 | 0.0292 | -0.2082 | 0.4642 | 0.915 | -0.4835 | 1 | 0.5745 | 0.9167 | -0.27 | 0.7087 | 0.4786 | 0.1973 |
| C-Type Ratio | 0.6087 | 0.6843 | -0.2262 | -0.2175 | -0.5 | 0.2999 | -0.4767 | 0.2138 | 0.669 | -0.8742 | 0.5745 | 1 | 0.4215 | -0.2106 | 0.5951 | 0.3065 | 0.0376 |
| A-Type Peak | 0.2006 | 0.2424 | -0.0698 | -0.0274 | -0.1198 | -0.0096 | -0.0736 | 0.3624 | 0.7213 | -0.2748 | 0.9167 | 0.4215 | 1 | -0.3461 | 0.6163 | 0.5227 | 0.3402 |
| A-Type Ratio | 0.0573 | 0.1415 | 0.1093 | 0.1617 | -0.4186 | -0.0494 | -0.1621 | -0.0051 | 0.0269 | -0.3143 | -0.27 | -0.2106 | -0.3461 | 1 | 0.0903 | -0.1305 | -0.2908 |
| Granule Size 10th Percentile (um) | 0.5107 | 0.599 | -0.3458 | -0.2574 | -0.2976 | 0.134 | -0.4025 | 0.2859 | 0.7082 | -0.6188 | 0.7087 | 0.5951 | 0.6163 | 0.0903 | 1 | 0.7788 | 0.4051 |
| Granule Size 50th percentile (um) | 0.3912 | 0.4054 | -0.5567 | -0.4873 | 0.1225 | 0.1877 | -0.2004 | 0.0089 | 0.3308 | -0.2895 | 0.4786 | 0.3065 | 0.5227 | -0.1305 | 0.7788 | 1 | 0.8825 |
| Granule Size 90th Percentile (um) | 0.2353 | 0.1871 | -0.5922 | -0.556 | 0.4047 | 0.2077 | 0.0002 | -0.2069 | -0.0436 | 0.0231 | 0.1973 | 0.0376 | 0.3402 | -0.2908 | 0.4051 | 0.8825 | 1 |

Table A.11. Correlations of sensory texture attributes to raw sweetpotato properties. Emboldened were the strongest significant correlations with the positive/negative relationship in parentheses and the R^2^ of a K-fold cross validation.

|  | | Sweetpotato Compositions (R^2^) | | | | | | | | | | | | |
| --- | --- | --- | --- | --- | --- | --- | --- | --- | --- | --- | --- | --- | --- | --- |
|  | | Starch | | Dry Matter | | Cell Wall Material | Glucose | Fructose | | | Sucrose | α-amylase | β-amylase | |
| Fracturability | | 0.163 | | 0.207 | | 0.224 | 0.268 | 0.344 | | | 0.007 | 0.009 | 0.082 | |
| Firmness | | 0.219 | | 0.260 | | 0.306 | 0.312 | 0.368 | | | 0.001 | 0.036 | 0.138 | |
| Moisture in the Mass | | 0.637 **(-, 0.426)** | | 0.635 **(-, 0.326)** | | 0.407 | 0.300 | 0.323 | | | 0.103 | 0.187 | 0.084 | |
| Mealiness | | 0.681 **(+, 0.557)** | | 0.664 **(+, 0.520)** | | 0.400 | 0.135 | 0.158 | | | 0.233 | 0.182 | 0.040 | |
| Adhesiveness | | 0.276 | | 0.203 | | 0.042 | 0.000 | 0.001 | | | 0.311 | 0.156 | 0.122 | |
| Cohesiveness | | 0.450 | | 0.339 | | 0.029 | 0.041 | 0.032 | | | 0.178 | 0.316 | 0.102 | |
| Smoothness | | 0.067 | | 0.118 | | 0.277 | 0.210 | 0.297 | | | 0.014 | 0.010 | 0.218 | |
| Particle Size | | 0.133 | | 0.058 | | 0.018 | 0.049 | 0.071 | | | 0.304 | 0.110 | 0.220 | |
| Rate of Breakdown | | 0.325 | | 0.364 | | 0.372 | 0.350 | 0.420 | | | 0.011 | 0.074 | 0.076 | |
| Fibrousness | | 0.090 | | 0.055 | | 0.013 | 0.086 | 0.061 | | | 0.052 | 0.047 | 0.035 | |
| Uniformity of Texture | | 0.015 | | 0.000 | | 0.066 | 0.069 | 0.130 | | | 0.235 | 0.094 | 0.131 | |
|  | Sweetpotato Starch Properties (R^2^) | | | | | | | | | | | | | |
|  | B-Type Peak Temp. | | B-Type Ratio | | C-Type Peak Temp. | C-Type Ratio | A-type Peak Temp. | | A-type Ratio | Granule Size 10^th^% | | Granule Size 50^th^% | | Granule Size 90^th^% |
| Fracturability | 0.027 | | 0.034 | | 0.030 | 0.016 | 0.052 | | 0.003 | 0.002 | | 0.016 | | 0.047 |
| Firmness | 0.006 | | 0.065 | | 0.013 | 0.036 | 0.024 | | 0.000 | 0.003 | | 0.027 | | 0.038 |
| Moisture in the Mass | 0.059 | | 0.360 | | 0.011 | 0.242 | 0.000 | | 0.027 | 0.082 | | 0.045 | | 0.012 |
| Mealiness | 0.179 | | 0.552 **(-, 0.443)** | | 0.043 | 0.373 | 0.002 | | 0.074 | 0.129 | | 0.019 | | 0.001 |
| Adhesiveness | 0.410 | | 0.454 | | 0.233 | 0.312 | 0.136 | | 0.062 | 0.300 | | 0.010 | | 0.045 |
| Cohesiveness | 0.340 | | 0.367 | | 0.206 | 0.306 | 0.158 | | 0.017 | 0.317 | | 0.074 | | 0.001 |
| Smoothness | 0.095 | | 0.003 | | 0.130 | 0.000 | 0.153 | | 0.001 | 0.028 | | 0.001 | | 0.021 |
| Particle Size | 0.579 **(-, 0.591)** | | 0.292 | | 0.361 | 0.230 | 0.257 | | 0.055 | 0.268 | | 0.015 | | 0.030 |
| Rate of Breakdown | 0.000 | | 0.102 | | 0.007 | 0.053 | 0.013 | | 0.001 | 0.003 | | 0.025 | | 0.039 |
| Fibrousness | 0.016 | | 0.101 | | 0.000 | 0.078 | 0.003 | | 0.027 | 0.006 | | 0.005 | | 0.002 |
| Uniformity of Texture | 0.334 | | 0.091 | | 0.205 | 0.084 | 0.191 | | 0.033 | 0.171 | | 0.036 | | 0.001 |

Table A.12. Summary of multivariate models for predicting sensory texture attributes from physicochemical raw sweetpotato properties. The best fit model was reported along with significant factors if R^2^>0.50. Percent contributions in bootstrapped forest models are reported next to factors and >50% were emboldened and underlined, >25% were emboldened, and listed were >10%.

| Sensory Texture | R^2^ | RASE | Significant Factors | | | |
| --- | --- | --- | --- | --- | --- | --- |
| Fracturability | 0.483 | 1.072 | NA |  |  |  |
| Firmness | 0.573 | 1.081 | **β-Amylase (50.1%)** | Starch (11.8%) | α-Amylase (11.7%) | |
| Moisture in the Mass | 0.656 | 0.918 | **Starch (63.6%)** | |  |  |
| Mealiness | 0.740 | 0.548 | **Starch (38.0%)** | B-Type Ratio (19%) | Cell Wall Material (13.0%) | |
| Adhesiveness | 0.544 | 0.446 | **B-Type Peak Temp. (37.1%)** | B-Type Ratio (18.5%) | C-Type Ratio (11.0%) | |
| Cohesiveness | 0.608 | 0.763 | **B-Type Peak Temp. (40.4%)** | **Starch (28.4%)** | α-Amylase (15.3%) | |
| Smoothness | 0.417 | 1.020 | NA |  |  |  |
| Particle Size | 0.522 | 0.962 | **B-Type Peak Temp. (43.5%)** | | |  |
| Rate of Breakdown | 0.537 | 1.239 | **β-Amylase (28.4%)** | Cell Wall Material (16.5%) | α-Amylase (15.3%) | Starch (14.0%) |
| Fibrousness | 0.195 | 0.654 | NA |  |  |  |
| Uniformity of Texture | 0.341 | 0.812 | NA |  |  |  |

Figure A.4. Baked sweetpotato maltose content in respect to starch content. Linear correlation calculation (Blue) excluded the β-amylase null genotypes (Orange).

Table A.13. Sugar composition (fresh weight basis) of 15 baked sweetpotato genotypes and perceived sweetness intensity as determined by a trained descriptive sensory analysis panel.

|  | Glucose (mg/g) baked | Fructose (mg/g) baked | Sucrose (mg/g) baked | Maltose (mg/g) baked | Sweetness Intensity^1^ |
| --- | --- | --- | --- | --- | --- |
| Beauregard | 11.89 ± 2.76 | 8.24 ± 2.38 | 41.16 ± 12.32 | 42.94 ± 6.58 | 5.39 ± 0.59 |
| Bonita | 9.58 ± 4.62 | 7.41 ± 2.57 | 30.81 ± 14.79 | 62.76 ± 3.58 | 4.76 ± 0.81 |
| Covington | 9.74 ± 2.51 | 5.94 ± 1.17 | 52.23 ± 4.99 | 36.8 ± 3.42 | 5.87 ± 0.26 |
| Dimbuka Bukalula | 2.36 ± 0.37 | 1.61 ± 0.21 | 39.66 ± 3.15 | 83.41 ± 10.17 | 4.73 ± 0.40 |
| Japanese (Kokei No. 14) | 6.08 ± 1.36 | 4.75 ± 1.33 | 37.22 ± 1.09 | 94.46 ± 14.22 | 5.86 ± 0.43 |
| NCMC16-0298 | 14.40 ± 1.61 | 9.66 ± 0.97 | 22.18 ± 1.13 | 60.68 ± 3.46 | 4.52 ± 0.36 |
| NC13-1027 | 6.27 ± 1.2 | 3.71 ± 0.66 | 57.32 ± 2.48 | 0.79 ± 0.7 | 4.81 ± 0.54 |
| NC15-0633 | 6.05 ± 0.41 | 3.44 ± 0.14 | 52.08 ± 0.81 | 30.62 ± 1.39 | 5.1 ± 1.16 |
| NC16-0613 | 3.97 ± 0.59 | 2.57 ± 0.52 | 47.3 ± 4.65 | 46.01 ± 12.95 | 3.79 ± 0.63 |
| NC17-0331 | 25.92 ± 3.22 | 15.58 ± 2.51 | 33.86 ± 3.29 | 19.54 ± 6.72 | 4.53 ± 0.53 |
| NCDM04-001 | 2.08 ± 0.59 | 0.91 ± 0.32 | 28.63 ± 2.64 | 1.75 ± 0.69 | 1.29 ± 0.29 |
| NCP13-0315 | 12.23 ± 0.8 | 7.59 ± 0.95 | 31.89 ± 4.32 | 52.68 ± 8.84 | 3.97 ± 0.62 |
| NCP16-0046 | 5.62 ± 0.43 | 3.79 ± 0.23 | 27.57 ± 2.15 | 88.11 ± 3.33 | 4.33 ± 0.36 |
| NCP16-0095 | 3.09 ± 0.78 | 1.69 ± 0.59 | 36.15 ± 3.38 | 108.17 ± 10.8 | 4.71 ± 0.87 |
| O'Henry | 12.39 ± 4.9 | 9.22 ± 2.38 | 22.4 ± 8.48 | 61.55 ± 3.15 | 4.38 ± 1.00 |

^1^ Average sweetness intensity determined by a trained descriptive sensory analysis panel and a 0-10 point intensity scale.

A

B

Figure A.5. The relationship between sugar contents and perceived sweetness intensity in baked sweetpotatoes. Panel A shows weak relationship to total sugar contents, and Panel B shows some improvement in the relationship after adjusting for sucrose sweetness equivalence (sucrose 1; glucose 0.64, fructose 1.2, and maltose 0.43) according to Shallenberger (1993).
